# Widespread but Divergent Drought Legacy Effects on Gross Primary Productivity Across Biomes

**DOI:** 10.1111/gcb.70541

**Published:** 2025-10-10

**Authors:** Xin Yu, René Orth, Markus Reichstein, Christian Reimers, Ulisse Gomarasca, Mirco Migliavacca, Dario Papale, Michael Bahn, Ana Bastos

**Affiliations:** ^1^ Department of Biogeochemical Integration Max Planck Institute for Biogeochemistry Jena Germany; ^2^ Department of Ecology Universität Innsbruck Innsbruck Austria; ^3^ Faculty of Environment and Natural Resources University of Freiburg Freiburg Germany; ^4^ Joint Research Centre European Commission Ispra Varese Italy; ^5^ Research Institute on Terrestrial Ecosystems (IRET) National Research Council of Italy (CNR) Monterotondo Italy; ^6^ Department for Innovation in Biological, Agro‐Food and Forest Systems (DIBAF) University of Tuscia Viterbo Italy; ^7^ Institute for Earth System Science and Remote Sensing Leipzig University Leipzig Germany

**Keywords:** drought, eddy covariance, gross primary productivity, hydraulic vulnerability, legacy effects, machine learning

## Abstract

Drought can impact terrestrial ecosystems concurrently but also lagged in time, that is, through legacy effects. Drought legacy effects have been identified in parameters such as tree radial growth or satellite‐based greenness. Evidence in ecosystem‐scale fluxes, for example, gross primary productivity (GPP), is emerging, but still limited to individual sites or specific regions. Based on GPP data at 76 long‐term (≥ 7 years) eddy‐covariance sites across climates and biomes, we found drought legacy effects for 26 out of 52 droughts, with magnitudes comparable to the drought concurrent effects and lasting typically up to 1 year following drought. These effects diverged in direction: 19 events led to reduced GPP in the following years (negative legacy effects), while 7 showed increased GPP (positive legacy effects). Forests experienced more negative legacy effects than non‐forest ecosystems. Legacy effects were more pronounced in forests with higher hydraulic vulnerability. Our findings demonstrate the global relevance of drought legacy effects on GPP and the need for their integration into drought impact assessments.

## Graphical abstract

Droughts can impact ecosystem CO_2_ uptake beyond their occurrence. By studying 76 flux towers worldwide, we found that half of the drought events we examined left lasting impacts on ecosystem CO_2_ uptake. These legacy effects usually lasted up to a year and were often beneficial for non‐forested ecosystems while harmful for forests, especially forests with greater hydraulic vulnerabilities. Our findings highlight the global significance of drought legacy effects on ecosystem CO_2_ uptake and underscore the importance of incorporating them into drought impact assessments.

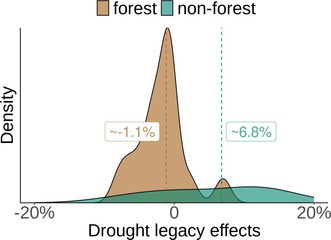

## 1 Introduction

Drought is a predominant driver of variability in the terrestrial ecosystem carbon cycle (Frank et al. 2015; Reichstein et al. 2013; Zscheischler et al. 2014) and has been suggested to cause temporary net carbon losses in ecosystems (Ciais et al. 2005; Gharun et al. 2020; Schwalm et al. 2012). Drought frequency and severity have been increasing in many regions, a trend expected to continue in the future (Intergovernmental Panel on Climate Change [IPCC] 2023). Thus, understanding the response of ecosystems to drought is crucial for understanding climate change impacts on the global carbon cycle (Piao et al. 2019).

Droughts are known to have strong immediate (concurrent) effects on gross primary productivity (GPP), such as those of severe droughts in Europe (Bastos et al. 2020; Ciais et al. 2005), China (Deng et al. 2021), the Amazon (Flack‐Prain et al. 2019), and North America (Schwalm et al. 2012). Concurrent impacts are typically associated with downregulation of photosynthetic activity (Grieco et al. 2020) and stomatal closure (Pirasteh‐Anosheh et al. 2016), or earlier leaf senescence that results in GPP decline (Widuri et al. 2020). Much less is known about whether and how droughts affect GPP in the seasons and years following a drought event. These so‐called drought legacy effects are commonly defined as alterations in ecosystem state or processes that occur after a drought has subsided (Müller and Bahn 2022). Current terrestrial biosphere models are not able to fully capture such legacy effects, thus failing to simulate the impacts of more frequent droughts (Bastos et al. 2021).

Drought legacy effects could result from multiple underlying mechanisms from individual to ecosystem scales (Müller and Bahn 2022). Drought‐induced structural and physiological changes such as smaller leaf area (Wu et al. 2018), xylem embolism (Anderegg et al. 2013; Sevanto et al. 2014), carbon reserve depletion (Adams et al. 2017), and tree mortality (Allen et al. 2015), as well as enhanced insect attacks (Migliavacca et al. 2009; Erbilgin et al. 2021), may lead to a reduction in GPP (negative legacy effects). Conversely, GPP can also be enhanced following drought (positive legacy effects). Positive legacy effects could be linked to changes in carbon allocation (Hagedorn et al. 2016), relief from competition for the surviving individuals due to the die‐off of their neighbors (Gessler et al. 2020), species shifts for grass and/or forest understory (Ru et al. 2023), or increased nutrient availability after drought (Hofer et al. 2017; Mackie et al. 2019).

Drought legacy effects have been widely reported in structural parameters, including tree radial growth (Anderegg et al. 2015; Huang et al. 2018) and vegetation greenness inferred from satellites (Wu et al. 2018, 2022). However, tree radial growth represents only a fraction of the carbon taken up by ecosystems (i.e., the amount allocated to radial growth) and satellite‐based greenness is a coarse proxy for carbon uptake potential. Notably, these parameters are largely decoupled from the actual dynamics of ecosystem carbon uptake (GPP) (Cabon et al. 2022; Hartmann et al. 2020; Kannenberg, Novick, Alexander, et al. 2019; Oddi et al. 2022) (GPP), which may undergo strong alterations not only in response to fluctuating environmental conditions but also during recovery from drought. Previous studies have provided emerging evidence of drought legacy effects on GPP, but this remains limited to several individual sites (Kannenberg, Novick, Alexander, et al. 2019; Pohl et al. 2023; Yu et al. 2022; Haberstroh et al. 2025) or satellite proxies over specific regions (Kannenberg et al. 2020), or have only focused on recovery dynamics—the duration aspect of legacy effects (He et al. 2018; Schwalm et al. 2017; Shao et al. 2024; Zhang et al. 2021). However, it remains unclear whether drought legacy effects on GPP are globally widespread, as well as what their direction, magnitude, and underlying drivers are.

**FIGURE 1:**
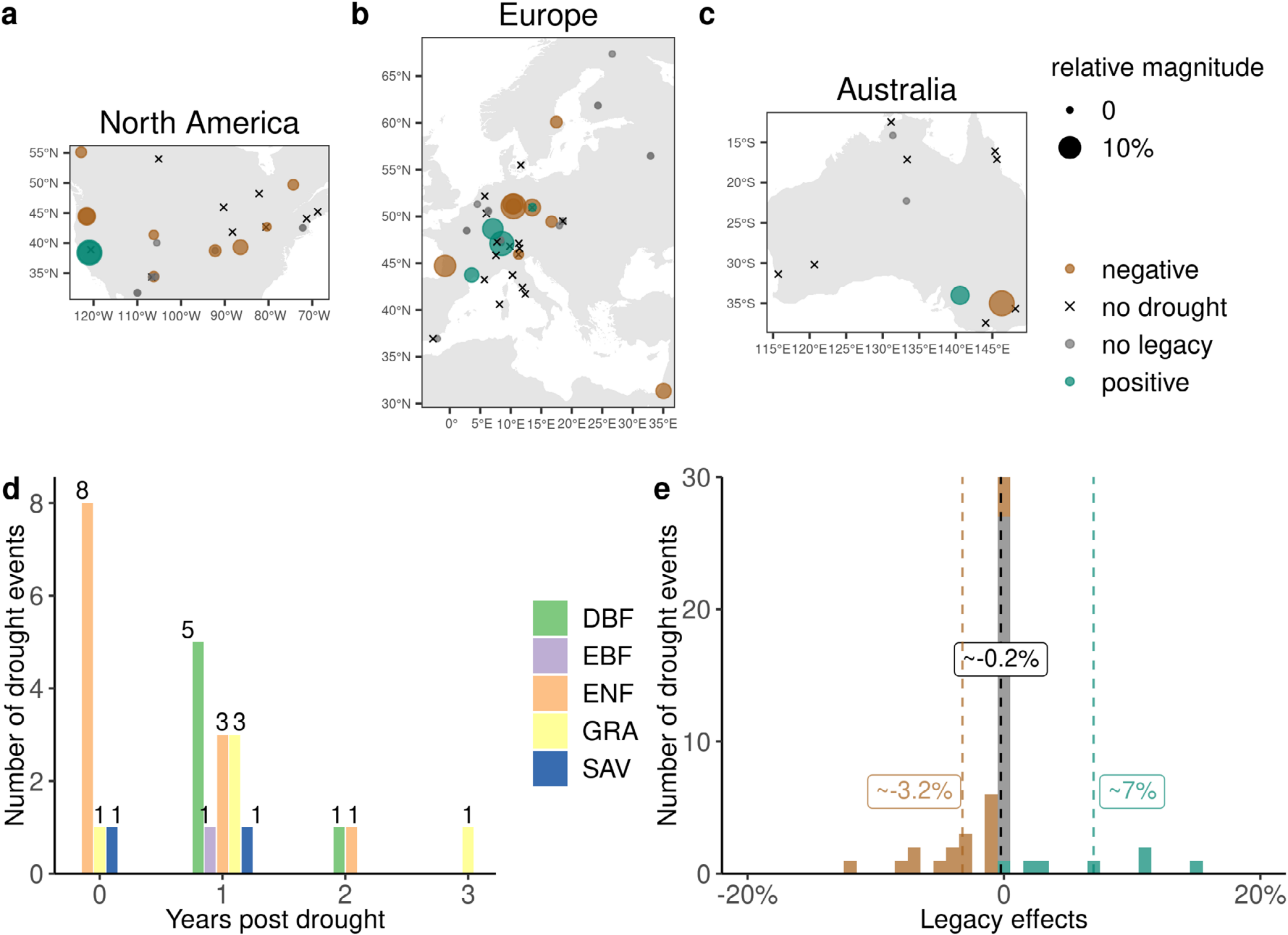
Drought legacy effects on gross primary productivity at investigated eddy‐covariance flux sites. (a) Cumulative drought legacy effects on GPP over identified legacy periods at sites in North America. The significance of legacy effects was assessed based on the Wilcoxon test when quantifying their durations. Green, brown, and grey points indicate sites where the drought caused positive, negative, or no significant legacy effects, respectively. Black crosses indicate sites that did not experience droughts during the study period. The size of the points corresponds to the magnitude of legacy effects expressed relative to their corresponding mean annual GPP based on the entire record of each site. (b) and (c) are similar to (a) but for sites in Europe and Australia. (d) Duration of drought legacy effects across biomes. The bars with the numbers above indicate the number of drought events in which significant legacy effects were detected in the post‐drought years and became nonsignificant after the legacy duration. Drought events of which legacy duration could not be quantified due to unavailable data record were excluded. Different colors indicate different plant functional types: DBF, deciduous broad‐leaf forests; EBF, evergreen broad‐leaf forests; ENF, evergreen needle‐leaf forests; GRA, grassland; SAV, savanna. (e) Frequency distribution of positive, negative, and no legacy effects across sites. The lines and labels show the mean for positive and negative legacy effects as well as the overall mean.

Here, we provide a large‐scale synthesis of drought legacy effects on GPP based on 76 long‐term (i.e., ≥ 7 years) eddy covariance measurement sites (Figure 1; Table S1) during 1992–2020 in forests, grasslands, shrublands, and savannas across Europe, North America, and Australia. We address the following questions: (1) How widespread are drought legacy effects on GPP across biomes? (2) What is the magnitude of drought legacy effects on GPP compared to their concurrent effects? (3) How common are negative and positive drought legacy effects on GPP in forests versus non‐forested ecosystems? And (4) How do climate, vegetation type, and plant traits alter drought legacies on GPP?

## 2 Materials and Methods

### 2.1 Eddy Covariance Data

The eddy covariance data (Table S1) used here belong to FLUXNET2015 (Pastorello et al. 2020), ICOS Warm Winter 2020 (Warm Winter 2020 Team and ICOS Ecosystem Thematic Centre 2022), AmeriFlux (Novick et al. 2018), and OzFlux (Beringer et al. 2016).

For each site, we used the following variables at the daily scale (Table 1): (i) gross primary productivity (GPP, gC m^−2^ d^−1^); (ii) latent heat fluxes (LE, W m^−2^); (iii) sensible heat fluxes (H, W m^−2^); (iv) air temperature (TA, °C); (v) vapor pressure deficit (VPD, hPa); (vi) incoming shortwave radiation (SW_IN, W m^−2^); (vii) net radiation (NETRAD, W m^−2^); (viii) precipitation (P, mm).

**TABLE 1:** Variables used in the analysis.

Variable	Abbreviation	Unit	Remark
Gross primary productivity	GPP	gC m^−2^ d^−1^	Target of the random forest model
Air temperature	TA	°C	Predictor of the random forest model
Vapor pressure deficit	VPD	hPa	Predictor of the random forest model
Incoming shortwave radiation	SW_IN	W m^−2^	Predictor of the random forest model
Water availability index	WAI	mm	Predictor of the random forest model
Precipitation	P	mm	Input for the WAI calculation
Evapotranspiration	ET	mm	Input for the WAI calculation
Evaporative fraction	EF	—	Drought definition
Latent heat flux	LE	W m^−2^	Input for the evaporative fraction
Sensible heat flux	H	W m^−2^	Input for the evaporative fraction
Net radiation	NETRAD	W m^−2^	Input for Aridity index

GPP was derived from the night‐time flux partitioning (GPP_NT; Reichstein et al. 2005) implemented in the ONEFLUX workflow (Pastorello et al. 2020). Although GPP based on the daytime partitioning method (GPP_DT; Lasslop et al. 2010) yields largely similar results (Figures S1–S4), we argue that GPP_NT is more appropriate for this study. Our machine learning model estimates drought legacy effects by relating GPP to meteorological variables. Since GPP_DT is derived using similar inputs (e.g., VPD), this overlap may introduce circularity and underestimate residuals during legacy periods. In contrast, GPP_NT relies mainly on air temperature, reducing this issue. This methodological difference is reflected in Figure S1, where the slope < 1 suggests weaker legacy effects in GPP_DT than GPP_NT.

Due to the missing data of LE, which is used for the water availability index (WAI) calculation, for each site, we removed the years with long‐period missing LE data from the analysis (see an example in Figure S5). Only sites with 7 or more years of available records are used for the purpose of having sufficient data for model training. The choice of 7 was based on the previous study (Yu et al. 2022), where we found 7 years were the minimum length needed to detect robust legacy effects, meaning that the estimates remain consistent when calculated using data records of 7 years or more. We performed a quality control to only retain data on days when more than 70% of measured and good quality gap‐fill data were available (QC > = 0.7). For DE‐Hai, a temperate forest in Germany, the energy‐balance‐corrected latent heat fluxes (LE_CORR) based on the assumption that the Bowen ratio is correct (Pastorello et al. 2020) in the dataset were used for the calculation of the water availability index. This was necessary because the non‐corrected LE underestimated the 2018 drought at that site (Yu et al. 2022). Since the partitioning of the energy imbalance between H and LE is highly site‐specific, applying corrections without site‐specific knowledge could introduce bias (Mauder et al. 2024). Therefore, we applied the correction only at DE‐Hai, where we have sufficient understanding of site conditions (Yu et al. 2022), and refrained from correcting other sites lacking such information.

For our analysis, we processed the eddy covariance and meteorological data as follows:

Growing season: We constrained the analysis to the growing season, a period defined by GPP being greater than 25% of the maximum value of the mean seasonal cycle of GPP (Panwar et al. 2023), which is determined based on a 7‐day centered moving average of daily GPP mean across all years for each site. For sites with nonstandard seasonal patterns—such as US‐Wkg, which exhibits a bimodal growing season, and US‐Var, where the growing season spans two calendar years—a smaller threshold of 10% was used to capture the full GPP seasonal cycle, ensuring both GPP peaks (or the full hydrological cycle) are included within the defined growing season (Figure S6).
(2) Anomalies: To analyze the drought‐related signals, we calculated anomalies of all variables by detrending and deseasonalizing the daily time series. Any significant long‐term linear trend detected by the Mann‐Kendall test (Kendall 1948) was removed from the original time series to avoid the influence of any factor causing the long‐term trend (e.g., CO_2_ fertilization effect (Zhan et al. 2024)). We calculated the mean seasonal cycle by taking the mean of each day of the year across all years for each site using the detrended time series.

Therefore, we used daily anomalies of variables in the growing season for each site in the subsequent analysis.

### 2.2 Abundance‐Weighted Hydraulic Traits

Following the approach in Anderegg et al. 2018, we combine the species composition of each site and the species‐specific traits from the TRY database (Kattge et al. 2020) to calculate the abundance‐weighted mean and standard deviation of plant hydraulic traits (Table S7). This method is considered effective, though it is not without limitations (Gomarasca et al. 2023; Musavi et al. 2016).

### 2.3 Water Availability Index

Soil moisture measurements are either not publicly available at some studied sites or only installed at the shallow layers at many of the studied sites. Missing deeper soil moisture signals might bias the drought impacts (Yu et al. 2022); therefore, we used a bucket‐type water balance model to estimate the water availability index (WAI), which captured the entire soil profile and purely used observed precipitation and evapotranspiration. The calculation is as follows:

(1) $${\mathrm{WAI}}_{0}={\mathrm{WAI}}_{\text{spin}-\mathrm{up}}$$

(2) $${\mathrm{WAI}}_{t}=\min\;\left({\mathrm{WAI}}_{\max},{\mathrm{WAI}}_{t-1}+P_{t}-{\mathrm{ET}}_{t}\right)$$

where WAI_0_ (mm) was the initial value of the calculation, and WAI_spin‐up_ (mm) was the final value of WAI from the warm‐up bucket model (Equation 2). We ran the bucket model five times through the first year to spin it up before the actual calculation across all studied years for each site. WAI_t‐1_ (mm) and WAI_t_ (mm) represented WAI at *t*‐1 and t time steps, respectively. *P*_t_ and ET_t_ were precipitation and evapotranspiration converted from LE using the heat of vaporization at the t time step, respectively. The maximum cumulative water deficit (CWD) experienced at each site was set as the bucket size (WAI_max_). CWD was calculated from cumulative differences between observed evapotranspiration and precipitation during time intervals characterized by positive cumulative net water loss from the soil (sum (ET—P)). We validated the WAI by comparing it to depth‐weighted soil water content (SWC) during the growing season at five sites, each with at least four measurement layers of known depth beyond 30 cm. The results showed a strong correlation between WAI and SWC (*r* = 0.71 ± 0.12; Figure S7), indicating that WAI effectively captures integrated soil moisture dynamics during the growing season at these sites and holds strong potential for broader application.

### 2.4 Drought Definition

Droughts occur in different ways across a continuum of systems (van Loon et al. 2024), and can be defined from the perspective of meteorology (precipitation deficit), hydrology (water shortage in streams or storage), agriculture (often related to low soil moisture), or ecology (related to plant water stress leading to, e.g., tree mortality (IPCC 2023)). Long‐lasting impacts from drought on ecosystems are expected to occur only when concurrent impacts negatively affect ecosystem functioning or its structure (Ruehr et al. 2019). Therefore, here, we followed a combined driver and impact‐based drought perspective (Reichstein et al. 2013; Smith 2011). Specifically, we are interested in drought events that lead to evidenced ecological impacts, traceable to anomalous meteorological conditions. The evaporative fraction (EF) has been used as a proxy for droughts in ecological studies (Fu et al. 2022; Graf et al. 2020; Schwalm et al. 2010). We calculated EF as follows:

(3) $$\mathrm{EF}=\frac{\mathrm{LE}}{\mathrm{LE}+H}$$

where *H* and LE correspond to sensible and latent heat fluxes, respectively. EF thus reflects the fraction of energy used for evaporation. For high values of EF, most of the available energy is used for evaporation (LE> > H), indicating that enough water at the surface is available, and it can flow unrestricted through the soil–plant‐atmosphere continuum. As available water declines, more energy is lost through sensible heat, thus EF declines. Beyond a critical threshold, plant stress can be identified by a sharp decline in EF as evaporation declines, for example, due to stomatal conductance decrease (Fu et al. 2022). Therefore, EF, on the one hand, indicates the overall water limitation of the ecosystem and, on the other hand, also captures the ecosystem's stress response to the water limitation.

In a preprocessing step, we removed unrealistic values (> 1 or < 0) of EF time series, and then, EF anomalies were calculated in the same way as other variables.

Drought events at each site were then defined as follows. First, we assessed the presence of drought conditions for each day. If the daily EF anomaly was below −1 *σ* of the entire data record, the day was flagged as experiencing drought. We verified that our estimates of legacy effects were robust even when based on different thresholds (Figure S8).

We identified a drought event as the period of at least 15 consecutive drought days (results are consistent based on different lengths, Figure S9). To avoid the effect of data gaps and noise, we considered drought events with small gaps in between, provided that these gaps were smaller than 4 days (results are consistent based on different lengths, Figure S10).

To ensure the low EF anomalies of identified drought events were caused by meteorological droughts, we further selected drought events with the traceable meteorological droughts based on the 90‐day Standardized Precipitation Evapotranspiration Index (SPEI_90_). Specifically, we selected drought events with at least 1 day of SPEI_90_ < −1 during the event or in the preceding 15 days, accounting for ecosystem response lag to meteorological drought. The selected threshold of SPEI does not lead to significant differences in legacy effects (Figure S11). The use of both gridded data and in situ measurements is because (1) the gridded data are too spatially coarse to match the footprint of eddy covariance sites but provides the long‐term climate background; (2) the in situ measurements are not long enough to capture the long‐term background but provide local drought signals.

The daily SPEI_90_ for each site was calculated using data of the nearest pixel to the site coordinate from ERA5‐land with a spatial resolution of 0.1° × 0.1° and the timespan of 1982–2022. The calculation of SPEI followed the methodology described in Vicente‐Serrano et al. 2010, but at the daily scale with an aggregation time of 90 days. The required inputs were precipitation and potential evapotranspiration (PET), which was calculated using the Priestley–Taylor equation (Priestley and Taylor 1972):

(4) $$\text{λPET}=1.26\frac{s\left(R_{n}-G\right)}{s+\gamma }$$

with *λ* being the latent heat of vaporization (J kg^−1^), *s* the slope of the Clausius‐Clapeyron curve relating air temperature with the saturation vapor pressure (Pa K^−1^), *R*_n_ the net radiation (W m^−2^), G the ground heat flux (W m^−2^), and *γ* the psychrometric constant (Pa K^−1^).

The drought event with the lowest negative cumulative EF anomaly (the strongest drought) was selected for the analysis of legacy effects for each site. The choice of only investigating the strongest drought at each site is a compromise of limited data record and the need for adequate data for model training. For sites with 14 years or longer records, we further selected the second strongest drought in order to consider a larger number of events in our analysis. Ultimately, we analyzed 52 drought events with durations of 35 ± 24 days across sites/events (Figure S12).

### 2.5 Quantification of Concurrent Drought Effects

Concurrent effects refer to the immediate effects of each drought event on GPP, which are calculated as the cumulative GPP anomalies during the corresponding drought periods. To compare across sites, concurrent effects for each drought event were normalized relative to the respective long‐term growing season mean GPP. The quality control introduced gaps in the data, and any gap during the drought periods would bias the concurrent effects. Therefore, we used the gap‐filled data from the dataset to calculate the concurrent effects.

### 2.6 Quantification of Legacy Effects

Since variability of environmental drivers can confound the detection of legacy effects when analysing post‐drought GPP dynamics, here we are following the methodology applied by Anderegg et al. (2015) and Yu et al. (2022). We quantify legacy effects as the departure of observed GPP from the expected GPP in the periods following a given drought, given the hydrometeorological conditions in that period. For a given time step (*t*), drought legacy effects on GPP (GPP_legacy,t_) correspond to the residuals of a random forest regression model that predicts potential GPP anomalies (GPP*_anom,t_) based on climatic drivers:

(5) $${\mathrm{GPP}}_{\text{legacy},t}={{\mathrm{GPP}}^{*}}_{\text{anom},t}-{\mathrm{GPP}}_{\text{anom},t}$$

where GPP_anom,t_ is the measured GPP anomaly. The random forest regression model used to predict GPP*_anom_,_t_ was fit separately for each site, trained on all days except legacy periods which are determined by an iterative strategy, gradually extending the duration until legacy effects were no longer statistically significant and using SW_IN_anom_, TA_anom_, VPD_anom_, WAI_anom_, and day of year (DOY) as predictors. Models achieved a satisfactory performance with the out‐of‐bag (OOB) score of 0.49 ± 0.10 across sites, and there was no significant relationship between OOB score and quantified legacy effects (Figure S13). This forms the basis to determine legacy effects based on the random forest model output. Random forest regression models can effectively learn the relationships between independent and dependent variables, irrespective of linear or nonlinear relationships, and furthermore considers interactions among independent variables (Ryo and Rillig 2017), which are more appropriate in this case, given the covariance between the predictors. To ensure the reliability of the training data, data flagged as low quality were excluded from the training, but used to estimate residuals (i.e., legacy effects). To reduce the influence of daily variability and outliers, we aggregated the daily legacy effect values into weekly averages.

To quantify the duration of the legacy effects, we used an iterative strategy to determine when legacy effects were no longer significant at an annual scale as follows:

First, we quantified legacy effects in the period following the drought event within the same growing season. Then, legacy effects were iteratively estimated in the following growing seasons until the effects across the growing season were not significantly (*p* > 0.05) different from zero (which we refer to as convergence) based on the Wilcoxon test. The duration of legacy effects was quantified as the number of growing seasons with significant legacy effects. For some sites with droughts closer to the end of the record, legacy effects did not converge due to unavailable data. These sites were still included for the analysis of magnitude (Figure 1a–c,e) but not for the analysis of duration (Figure 1d) of legacy effects. For the sites that registered two drought events, if the 1st drought event could not converge until the 2nd drought event, we discarded the 1st drought event from the analysis. We provided a case example (Figures S14 and S15) to illustrate the non‐legacy, drought, and legacy periods and the quantification of the duration of legacy effects.

After quantifying the legacy duration, drought legacy effects on GPP considering uncertainty (GPP_legacy_unc,t_) were quantified in Equation (6) as follows:

(6) $${\mathrm{GPP}}_{\text{legacy}\_\mathrm{unc},t}=\left\{\begin{array}{c} \max\left(\text{median}\left({\mathrm{GPP}}_{\text{anom},t}^{*}\right)-{\mathrm{GPP}}_{\text{anom},t}-p95\left(X_{\text{uncertainty}}\right),0\right),\\ \text{if median}\left({\mathrm{GPP}}_{\text{anom},t}^{*}\right)>{\mathrm{GPP}}_{\text{anom},t}\\ \min\left(\text{median}\left({\mathrm{GPP}}_{\text{anom},t}^{*}\right)-{\mathrm{GPP}}_{\text{anom},t}-\text{p5}\left(X_{\text{uncertainty}}\right),0\right),\;\\ \text{if median}\left({\mathrm{GPP}}_{\text{anom},t}^{*}\right)<{\mathrm{GPP}}_{\text{anom},t} \end{array}\right.$$

GPP*_anom,t_ and GPP_anom,t_ were the same as those in Equation (5). *X*_uncertainty_ was the distribution of model uncertainties. p5 and p95 were the 5th and 95th percentiles of the distribution. *X*_uncertainty_ at the corresponding day of the year (i.e., 366 distributions for 366 days of year) was quantified using a leave‐one‐out strategy (Figure S16): in the training phase of the predictive model, we excluded each of the non‐legacy years from the training dataset and predicted the GPP*_anom,t_ in that year. This procedure was iteratively repeated for all non‐legacy years in order to estimate a distribution of model residuals (*X*_uncertainty_) for all leave‐one‐out iterations, which reflect model uncertainties. GPP*_anom,t_ in the legacy duration was quantified at each of the iterations, and we took the median of these values, median (GPP*_anom,t_), for the further calculation. We strictly selected significant legacy effects only when the difference between median (GPP*_anom,t_) and GPP_anom,t_ is below or above the uncertainty range, given by the 5%–95% of the model residuals to ensure that the residuals reflect true legacy effects rather than prediction errors.

We integrated significant legacy effects during the legacy duration as cumulative legacy effects (GPP_legacy_cumu_) and normalized them by long‐term mean total GPP over the growing season for comparison across sites.

(7) $${\mathrm{GPP}}_{\text{legacy}\_\text{cumu}}=\sum_{t=1}^{n}{\mathrm{GPP}}_{\text{legacy}\_\mathrm{unc},t}$$

where *n* is the length of the legacy period.

The 2006 drought results in DE‐Tha, a temperate forest in Germany, were removed in the further analysis because a strong windthrow on the 18th of January 2007 caused by the cyclone “Kyrill” led to a large reduction in the entire growing season of 2007 (Spank 2010).

We found that GPP residuals during legacy years were significantly different from zero, while those in the year following the legacy period were not statistically significant (Figure S17). This pattern supports the existence of legacy effects, indicating that the impacts of drought persist beyond the event itself but eventually subside. To further assess the robustness of these effects, we compared GPP residuals during legacy years with model uncertainty at each site (Figure S18). In most cases, the residuals lay outside the interquartile range (25th–75th percentile) of model uncertainty, reinforcing the reliability of the observed legacy effects. Moreover, our method offers two key advances over previous studies (Anderegg et al. 2015; Kannenberg, Novick, Alexander, et al. 2019; Kannenberg, Novick, and Phillips 2019; Yu et al. 2022): it explicitly quantifies the duration of legacy effects, and it captures shifts in climate sensitivity, as detailed in Section *Climate Sensitivity Shifts* and illustrated in Figure S19 and Table S2.

### 2.7 Climate Sensitivity Shifts

Residual‐based approaches to estimate legacy effects (Anderegg et al. 2015; Yu et al. 2022) relied on the assumption that a changed sensitivity to climatic drivers would be indicative of changes in the functioning of ecosystems during post‐drought periods (Bastos et al. 2020, 2021; Wu et al. 2022), for example, due to hydraulic damage. Here, we analyzed whether this assumption holds by separately training two random forest models for non‐legacy and legacy periods for each site in which we detected significant legacy effects. We then evaluated the contribution of each observation of each predictor to the prediction of GPP_anom_ by calculating Shapley Additive Explanations (SHAP (Lundberg and Lee 2017)). Based on the Shapley values from cooperative game theory (Shapley 1953), SHAP assigns each observation of each predictor an importance value for the prediction of GPP_anom_, quantifying how much each observation of each predictor contributes, positively or negatively, to the prediction. To address feature dependence, we applied a conditional SHAP approach that models inter‐feature relationships during attribution estimation (Aas 2021). For each predictor, we fitted two response curves of SHAP to the predictor for legacy and non‐legacy periods separately based on LOESS (locally estimated scatterplot smoothing) with a span of 1. To minimize the influence of differing predictor ranges between the legacy and non‐legacy periods, we calculated SHAP values only within the common range shared by both periods for each predictor. Furthermore, to control for differences in sample size, we randomly subsampled the data in non‐legacy periods to match the sample size of legacy periods when training the random forest model. We checked how much the 95% confidence interval of the fitted two curves overlapped (Figure S19). A low overlap percentage indicates a shift in the response of GPP_anom_ to the respective predictor.

We found that for the drought events that lead to significant legacy effects, there was a shifted climatic dependence of GPP_anom_ during legacy periods relative to non‐legacy periods. The climatic dependence shift we found (Figure S19; Table S2) confirmed this assumption and further validated the drought legacy effects we quantified.

### 2.8 Causal Analysis

To explain the strong variability of legacy effects in space (i.e., across sites), we examined the role of the potential drivers listed in Table S3. These variables include vegetation type (forest vs. non‐forest), drought characteristics (timing, duration, and intensity), concurrent impact peak, concurrent effects (for definition refer to Table S3), and pre‐drought and post‐drought water limitation, as well as background climate conditions (aridity, mean temperature, precipitation seasonality, and temperature seasonality) and satellite‐derived rooting depth (Stocker et al. 2023). We used three different kernel‐based (un)conditional nonlinear independence test methods, which are Hilbert‐Schmidt independence criterion (HSIC) permutation test (Gretton et al. 2005), HSIC gamma (Gretton et al. 2005), and Kernel‐based Conditional Independence test (KCIT (Zhang et al. 2012)) to explore the relationships between these drivers and legacy effects. The analysis was done using the *kpcalg* (Verbyla et al. 2017) and *RCIT* (Strobl et al. 2019) packages in R 4.0.3 (R Core Team 2021). These independence test methods were used here because the relationships between these drivers and legacy effects were expected to be highly nonlinear, and they were able to account for nonlinearity while classical correlation and regression might not.

First, an unconditional independence test (see Tables S4 and S5) was adopted to explore legacy effects' dependence on individual potential drivers separately. For any potential driver, if at least two out of the three methods yield a *p*‐value of less than 0.05, this potential driver was selected as a candidate driver on which legacy effects were unconditionally dependent.

After the unconditional independence test, a conditional independence test should be used to find the causal structure among the candidate drivers if more than two are found. However, we only found one candidate driver among all potential drivers (see Tables S4 and S5); therefore, the selected candidate driver was identified as the causal driver.

We provided an example (Figure S20) to illustrate unconditional and conditional independence tests.

## 3 Results and Discussion

### 3.1 Widespread but Divergent Drought Legacy Effects on Gross Primary Productivity

We found significant legacy effects on GPP in 26 out of 52 drought events (Figure 1a–c) at 43 long‐term (≥ 7 years) eddy covariance sites distributed across North America, Europe, and Australia. For the 26 drought events for which legacy duration was quantified (see Section 2), legacy effects lasted up to 1 year after droughts ended in 23 events, and 2–3 years in three events (Figure 1d). Furthermore, we found a divergent pattern in the sign of legacy effects, with negative effects being more common: 19 drought events leading to negative effects (i.e., long‐lasting significant reduction in GPP), and 7 drought events to positive ones (i.e., long‐lasting significant enhancement in GPP; Figure 1e), but with comparable relative magnitude. On average, legacy effects led to a 0.2% reduction in GPP relative to the long‐term mean, with negative legacy effects resulting in a 3.2% reduction in GPP, while positive effects caused a 7.0% increase in GPP (Figure 1e).

The magnitude (~−1.2%; see Figure S21a) and duration (~1 year; see Figure 1d) of legacy effects on GPP that we found for forest sites were smaller and shorter than the legacy effects previously reported for tree radial growth (Anderegg et al. 2015), which implied a ~9% decrease in year one and ~5% in year two after drought and lasted up to 4 years. These differences in magnitude and duration could be due to a partial decoupling of GPP and radial growth due to shifts in carbon allocation (Cabon et al. 2022; Hartmann et al. 2020; Kannenberg, Novick, Alexander, et al. 2019; Oddi et al. 2022; Trugman et al. 2018). Based on satellite‐based vegetation greenness, previous studies reported drought legacy effects of 1 year or less for grasslands (Wu et al. 2018, 2022), which are consistent with our results, and are in line with previous observations that vegetation greenness closely reflects variations in grassland productivity (Hu, Piao, et al. 2022).

The drought legacy effects on GPP reported here could be due to a range of mechanisms. Negative legacy effects could be caused by different drought‐induced structural and physiological changes such as reduced leaf area (Wu et al. 2018) and xylem embolism (Sevanto et al. 2014). This might result in different persistent reductions in ecosystem productivity following droughts. When including the enhanced vegetation index, a proxy of canopy leaf area, as an additional predictor in the statistical model, we were able to better match GPP anomalies during the post‐drought periods (Figure S22). This suggests that canopy changes partly explain the identified negative legacy effects. Furthermore, we found that productivity can also be enhanced following drought (positive legacy effects), which might be due to changes in carbon allocation (Hagedorn et al. 2016), or species shifts in grasslands and/or forest understory (Ru et al. 2023). Furthermore, positive drought legacy effects on productivity could also be due to increased nutrient availability after drought inducing higher leaf nitrogen concentrations, especially for grasslands (Hofer et al. 2017; Mackie et al. 2019; Roy et al. 2016). For example, the positive legacy effects after the 2004 drought at US‐Var, a semiarid grassland in the US (Figure S23), coincide with reports of higher than‐usual leaf nitrogen concentrations (Ma 2012). By contrast, the effects of enhanced nutrient availability on trees might be less pronounced as they (1) follow a more resource‐conservative/slow‐growth strategy than most grassland species and (2) dilute any potential effects of enhanced nutrient availability through their larger biomass. While the occurrence of positive drought legacy effects has been previously discussed in the literature (Ingrisch et al. 2023; Roy et al. 2016; Ru et al. 2023), our study provides large‐scale evidence for the prevalence of positive legacy effects on GPP.

### 3.2 Distribution of Drought Legacy Effects on Gross Primary Productivity

**FIGURE 2:**
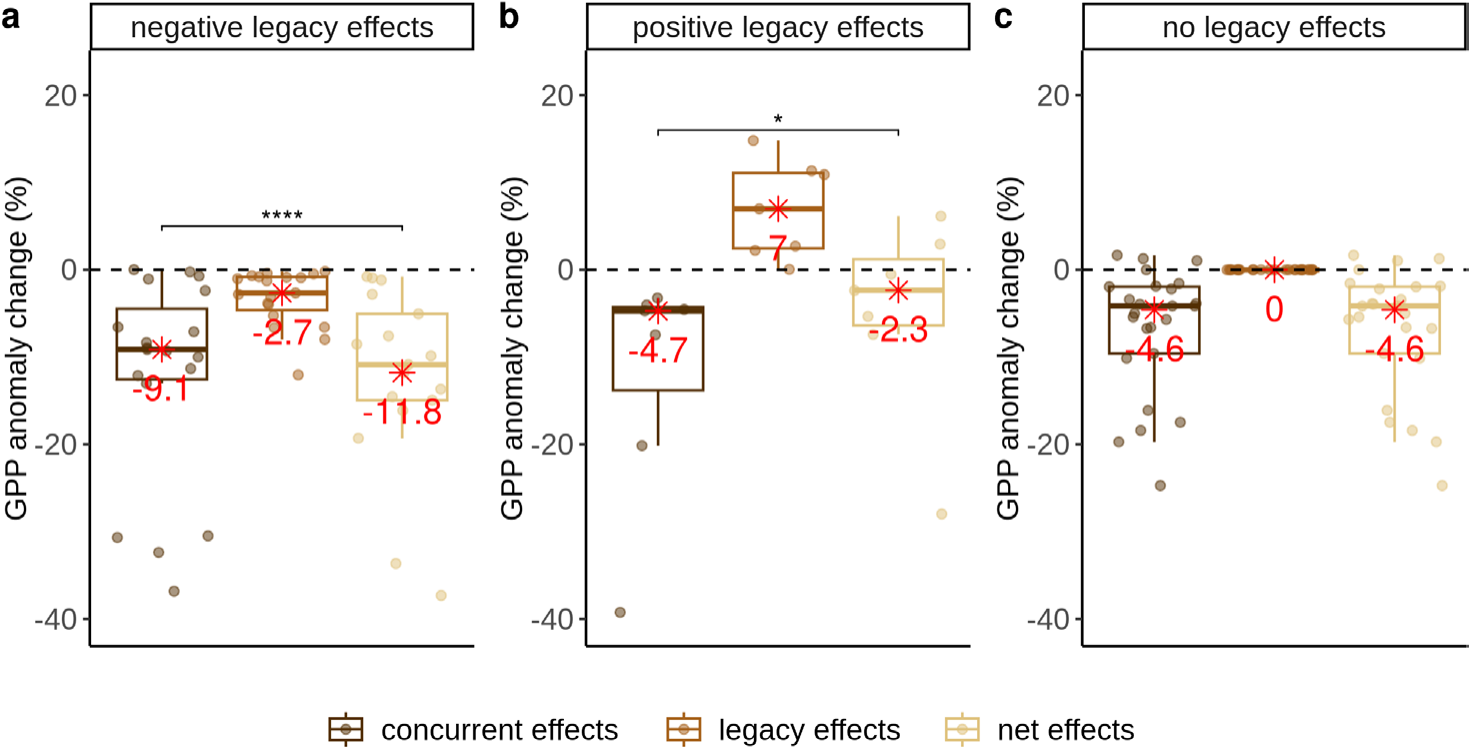
Comparison of the magnitude of concurrent, legacy, and net effects of drought on GPP across biomes (*N* = 52). Net effects are the sum of concurrent and legacy effects of drought events. Each point indicates the percent change in cumulative GPP anomalies induced by concurrent, legacy, or net effects of drought relative to the long‐term mean GPP based on the entire record at the respective site. The three panels summarize the drought events leading to (a) negative, (b) positive, and (c) no legacy effects, respectively. The significance labels indicate whether the difference in the mean of concurrent and net effects across sites is statistically significant (*p < 0.05; ****p < 0.0001), based on a paired two‐sample Wilcoxon test. The red asterisk and associated numbers are the median values of the corresponding boxplots.

In a next step, we compared drought legacy effects to concurrent drought impacts on GPP (Figure 2) based on the cumulative GPP anomalies (GPP_anom_) over the drought and legacy periods (see Section 2). We found that cumulative drought legacy effects were comparable with drought concurrent effects (Figure 2). Negative legacy effects (−2.7%) accounted for approximately 30% of the corresponding concurrent effects (−9%), thereby amplifying the overall net negative impacts of droughts on GPP (−12.7%). By contrast, positive legacy effects (7%) compensated for the concurrent effects (−4.7%), resulting in a small net effect (−2.3%). However, a further analysis conducted separately across biomes revealed that these amplification or compensation effects were only significant for evergreen needle‐leaf forests (Figure S24).

### 3.3 Forests Experience More Negative Legacy Effects Than Non‐Forest Ecosystems

**FIGURE 3:**
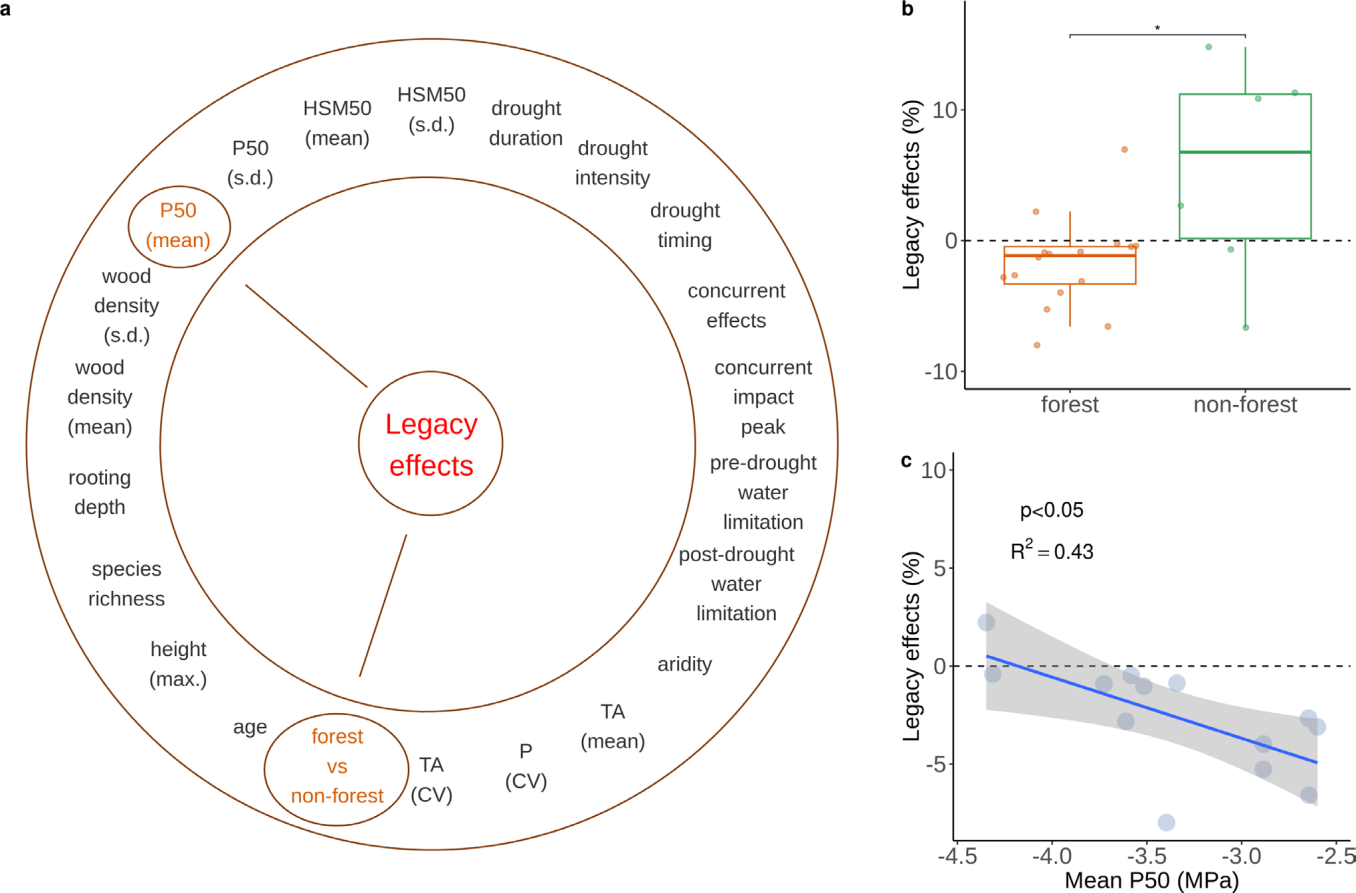
The factors modulating the spatial variability of drought legacy effects. (a) Causal link between potential explanatory factors and legacy effects across droughts which led to significant legacy effects and of which duration of legacy effects were quantified. All factors considered are shown in the outer circle and identified dependent factors are shown in brown, while independent factors are shown in black (see Section 2). SD and CV represent standard deviation and coefficient of variation, respectively. See Table S3 for the detailed definition of all potential explanatory factors. (b) Comparison of drought legacy effects on GPP between forests and non‐forests. The significance label indicates the difference in legacy effects across forest and non‐forest sites is statistically significant (*p < 0.05), based on the independence test (see Section 2). (c) Stronger legacy effects are associated with ecosystems with less negative mean P50 (the water potential at 50% loss of hydraulic conductivity). P50 was upscaled from species weighted by their relative abundance in the footprint of the respective EC tower. The blue line with the grey area indicates the linear regression line and associated uncertainty. One potential outliner was removed from the linear fitting based on Mahalanobis Distance (Figure S25).

To explain the variability in drought legacy effects across sites, we analyzed the causal relationships between legacy effects and a group of variables relevant to drought resistance and resilience for which data is available across sites. We found a significant dependence of legacy effects on vegetation type (i.e., forest vs. non‐forest; Table S4). Forests showed systematically more negative legacy effects than non‐forest ecosystems (Figure 3b). We hypothesize that the less negative effects in non‐forest ecosystems are due to rapid compensatory growth, which has repeatedly been observed in grasslands (Luo et al. 2023; Ru et al. 2023; Schärer et al. 2023). However, it is important to note that positive legacy effects were not observed in all studied non‐forest ecosystems. Future work should test the generality of such compensatory effects in non‐forest ecosystems and elucidate their drivers, which could be extrinsic ones, for example, drought intensity (Ingrisch et al. 2023; Oram et al. 2023) and intrinsic ones, for example, the functional attributes of species related to regeneration capacities such as belowground storage, drought sensitivity of apical meristems, and modularity.

### 3.4 Hydraulic Vulnerability Regulates Drought Legacy Effects in Forests

We then focused on forest ecosystems and examined the spatial variability of legacy effects in forests with additional forest‐specific explanatory variables compared to the analyses on all biomes. These additional variables (see Table S3 for a more detailed definition) included forest mean age, canopy maximum height, species richness (Table S6), and abundance‐weighted mean and standard deviation of wood density, water potential at 50% loss of hydraulic conductivity (P50), and hydraulic safety margin (HSM50, as the difference between P50 and the minimum water potential experienced, Table S7).

We found significant dependences of legacy effects on mean P50 (see Section 2, Table S5). Legacy effects were more prevalent in forests with less negative mean P50 (Figure 3c). This could be because forests with less negative mean P50 have higher embolism susceptibility, thereby causing more hydraulic loss induced by droughts. Therefore, repairing the hydraulic loss requires more resources and time (Ruehr et al. 2019), which results in stronger legacy effects. This finding aligns with Kannenberg, Novick, and Phillips (2019), who reported that more severe hydraulic damage—associated with less negative P50 values—led to delayed drought recovery. By contrast, Anderegg et al. (2018) suggested that greater diversity in HSM50 is linked to reduced concurrent drought impacts. However, we did not find a significant relationship between hydraulic trait diversity and drought legacy effects. Given the limited sample size in our study, this discrepancy cannot be conclusively attributed to differences between concurrent and legacy drought impacts, nor can the influence of trait diversity be ruled out. Overall, our results contribute to the growing body of evidence that plant hydraulic traits are key determinants of ecosystem responses in the post‐drought period (González de Andrés et al. 2021; Hu, Xiang, et al. 2022; Fatecha et al. 2025). Additionally, Song et al. (2022) showed that drought recovery in conifer species is driven by non‐hydraulic leaf traits, particularly leaf lifespan. Since data on non‐hydraulic leaf traits are limited, we are unable to assess their role in drought legacy effects. We therefore recommend further research to explore this potentially important aspect once more non‐hydraulic leaf traits data become available.

Management practices and disturbances such as thinning (Saunders et al. 2012), harvest (Paul‐Limoges et al. 2015), fire (Beringer et al. 2007), and grazing (Wall et al. 2020) influence stand age and age structure in forests (Au et al. 2022; Musavi et al. 2017) and can thereby alter GPP and the legacy effects. The relatively small available sample size (27 events) might explain why no significant causal link was found for some of the factors considered here, for example, species richness and canopy height. For other factors, in particular management, disturbances, and age structure, lack of data for most sites precluded the analysis of their potential effects.

## 4 Conclusions

Our global analysis of long‐term eddy‐covariance flux measurements suggests a high prevalence of significant drought legacy effects, which can manifest as either negative or positive, that is, reduction or enhancement of GPP typically in 1 year following drought, respectively. While the occurrence of positive drought legacy effects has been previously discussed in the literature, our study provides large‐scale evidence for the prevalence of positive legacy effects on GPP. The GPP anomalies from legacy effects are generally comparable to the concurrent GPP reductions during drought events, such that concurrent impacts are either amplified or buffered by legacies. Forests show more prevalent negative legacy effects than non‐forest ecosystems, and these were more pronounced when composed of tree species with higher hydraulic vulnerability (P50). Our findings demonstrate that drought legacy effects on GPP are smaller and shorter than legacy effects previously reported on tree radial growth and vegetation greenness. We hypothesize that shifts in carbon allocation cause this partial decoupling of GPP, radial growth, and greenness.

Our results highlight the need for deeper investigation into the mechanisms driving drought legacies, considering both plant‐related (e.g., carbon allocation and hydraulic traits) and soil‐related (soil properties, nutrient cycling) factors. Coordinated measurements across ecosystem monitoring networks would greatly enhance our understanding of ecosystem resilience under increasing drought stress. Additionally, the high variability within plant functional types underscores the importance of resolving structural and functional diversity in future studies.

## Author Contributions

**Xin Yu:** conceptualization, data curation, formal analysis, investigation, methodology, software, validation, visualization, writing – original draft, writing – review and editing. **René Orth:** conceptualization, funding acquisition, methodology, supervision, validation, visualization, writing – review and editing. **Markus Reichstein:** conceptualization, funding acquisition, methodology, resources, supervision, validation, visualization, writing – review and editing. **Christian Reimers:** methodology, validation, visualization, writing – review and editing. **Ulisse Gomarasca:** validation, visualization, writing – review and editing. **Mirco Migliavacca:** validation, visualization, writing – review and editing. **Dario Papale:** funding acquisition, validation, writing – review and editing. **Michael Bahn:** conceptualization, funding acquisition, methodology, supervision, validation, visualization, writing – review and editing. **Ana Bastos:** conceptualization, funding acquisition, methodology, resources, supervision, validation, visualization, writing – original draft, writing – review and editing.

## Conflicts of Interest

The authors declare no conflicts of interest.

## Supporting information


**Figures S1–S25.** gcb70541‐sup‐0001‐FigureS1‐S25.pdf.

**Tables S1–S7.** gcb70541‐sup‐0002‐TableS1‐S7.pdf.

## Data Availability

The data used in this study are publicly available in the following datasets: the FLUXNET2015 (https://fluxnet.fluxdata.org/data/fluxnet2015‐dataset/), the ICOS Warm Winter 2020 (https://www.icos‐cp.eu/data‐products/2G60‐ZHAK), the AmeriFlux FLUXNET (https://ameriflux.lbl.gov/introducing‐the‐ameriflux‐fluxnet‐data‐product/), and the OzFlux (https://data.ozflux.org.au/home.jspx) datasets. Code Availability: The code is available at https://doi.org/10.5281/zenodo.17137263.
