# Supplementary material for: Widespread but Divergent Drought Legacy Effects on Gross Primary Productivity Across Biomes
Source: Glob Chang Biol. 2025 Oct 10;31(10):e70541. doi: 10.1111/gcb.70541 (PMC12514344; doi:10.1111/gcb.70541)
Supplement: Supplementary file 1 — Figures S1–S25. gcb70541‐sup‐0001‐FigureS1‐S25.pdf. [file GCB-31-e70541-s002.pdf]

Contents in this file include:

Supplementary Figures 1-25

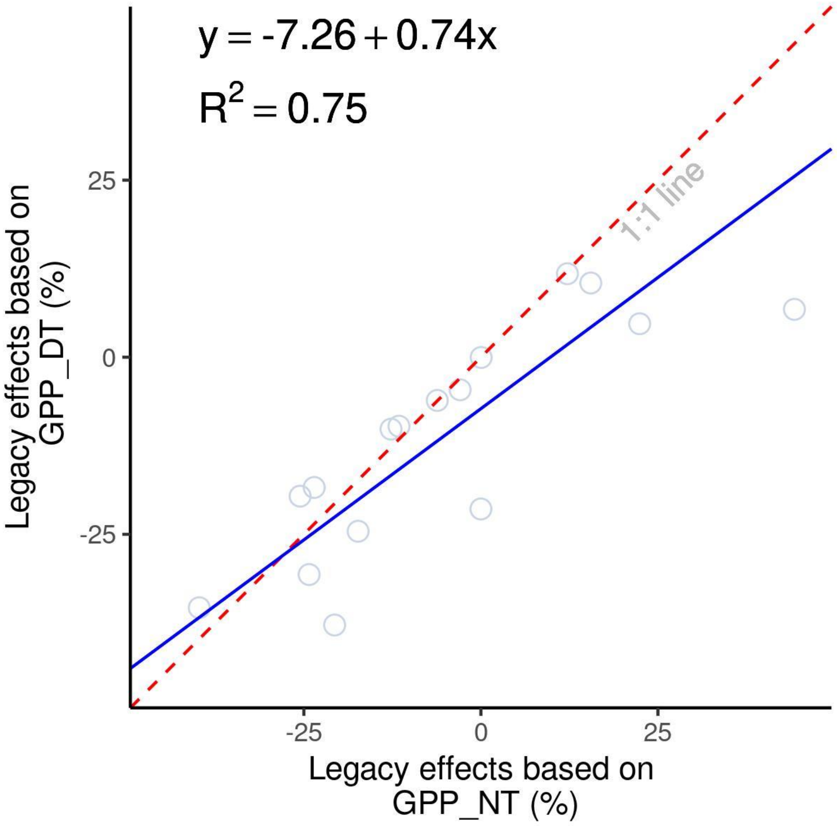

Figure S1. The comparison of legacy effects on GPP based on nighttime (GPP_NT) and daytime (GPP_DT) partitioning methods. The blue line is the fitting line based on Reduced Major Axis regression. Only the drought legacy effects detected by both GPP_NT and GPP_DT are shown here. To avoid the influence of the different uncertainties of GPP_NT and GPP_DT, the legacy effects without removing the uncertainty are shown here.

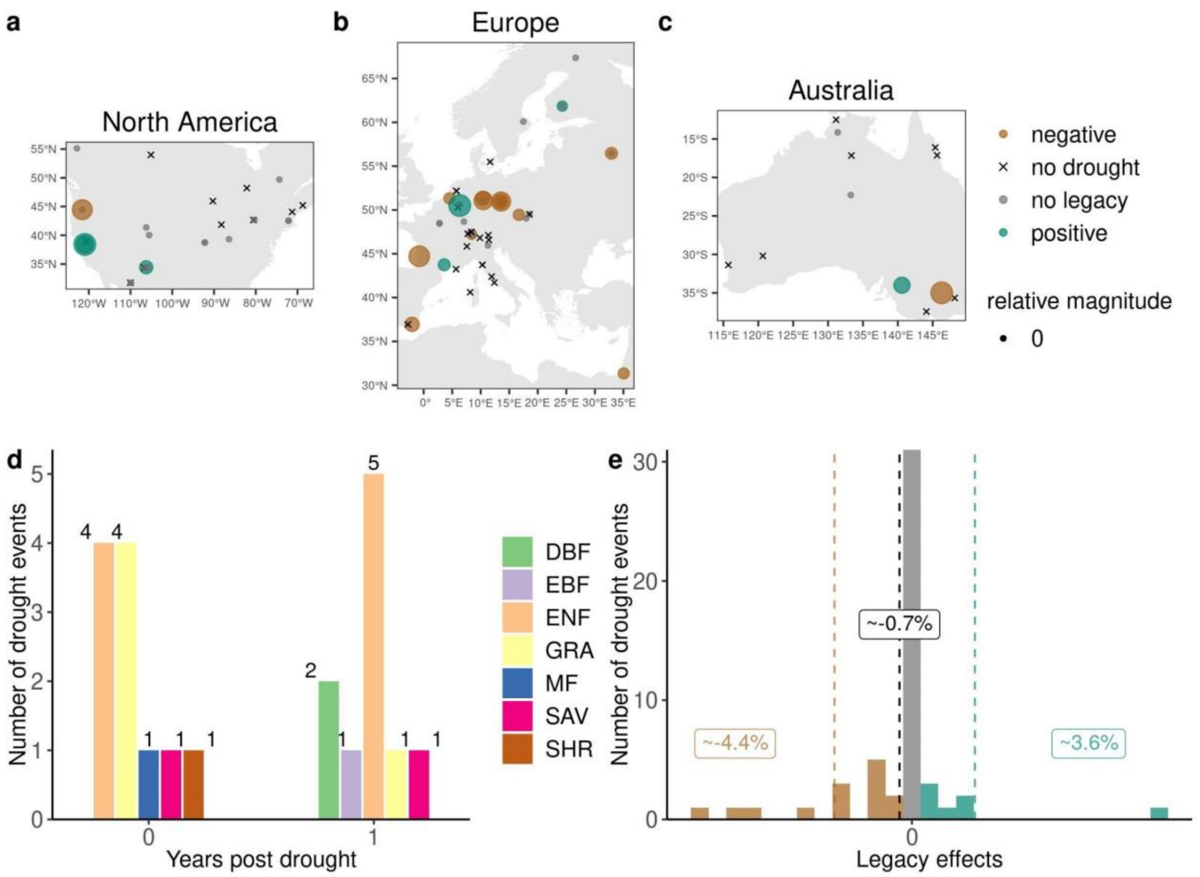

Figure S2. Drought legacy effects on gross primary productivity based on the daytime partitioning method at the investigated eddy-covariance flux sites. a) Cumulative drought legacy effects on GPP over identified legacy periods at sites in North America. The significance of legacy effects was assessed based on the Wilcoxon test when quantifying their durations (see Text S5). Green, brown, and grey points indicate sites where the drought caused positive, negative, or no significant legacy effects, respectively. Black crosses indicate sites that did not experience droughts. The size of the points corresponds to the magnitude of legacy effects expressed relative to their corresponding mean annual GPP based on the entire record of each site. **b)** and **c)** are similar to **a)** but for sites in Europe and Australia. **d)** Duration of drought legacy effects across biomes. The bars with the numbers above indicate the number of drought events in which significant legacy effects were detected in the post-drought years and became non-significant after the legacy duration. Drought events for which legacy duration could not be quantified due to unavailable data record were excluded. Different colors indicate different plant functional types: DBF, deciduous broad-leaf forests; EBF, evergreen broad-leaf forests; ENF, Evergreen needle-leaf forests; GRA, grassland and SAV, savanna. **e)** Frequency distribution of positive, negative, and no legacy effects across sites. The lines and labels show the mean for positive and negative legacy effects as well as the overall mean.

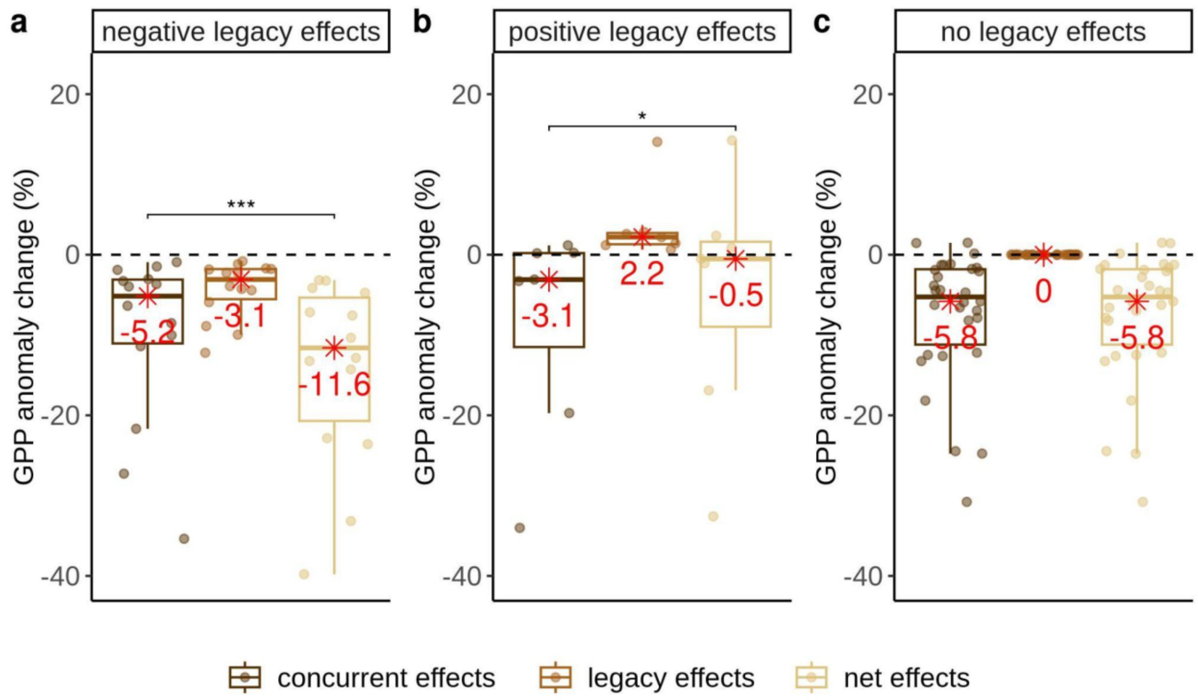

Figure S3. Comparison of the magnitude of concurrent, legacy and net effects of drought on GPP based on the daytime partitioning method across biomes (N=53). Net effects are the sum of concurrent and legacy effects of drought events. Each point indicates the percent change in cumulative GPP anomalies induced by concurrent, legacy, or net effects of drought relative to the long-term mean GPP based on the entire record at the respective site. The three panels summarize the drought events leading to **a)** negative, **b)** positive, and **c)** no legacy effects, respectively. The significance labels indicate whether the difference in the mean of concurrent and net effects across sites is statistically significant (* $p < 0.05$; *** $p < 0.001$), based on a paired two-sample Wilcoxon test. The red asterisk and associated numbers are the median values of the corresponding boxplots.

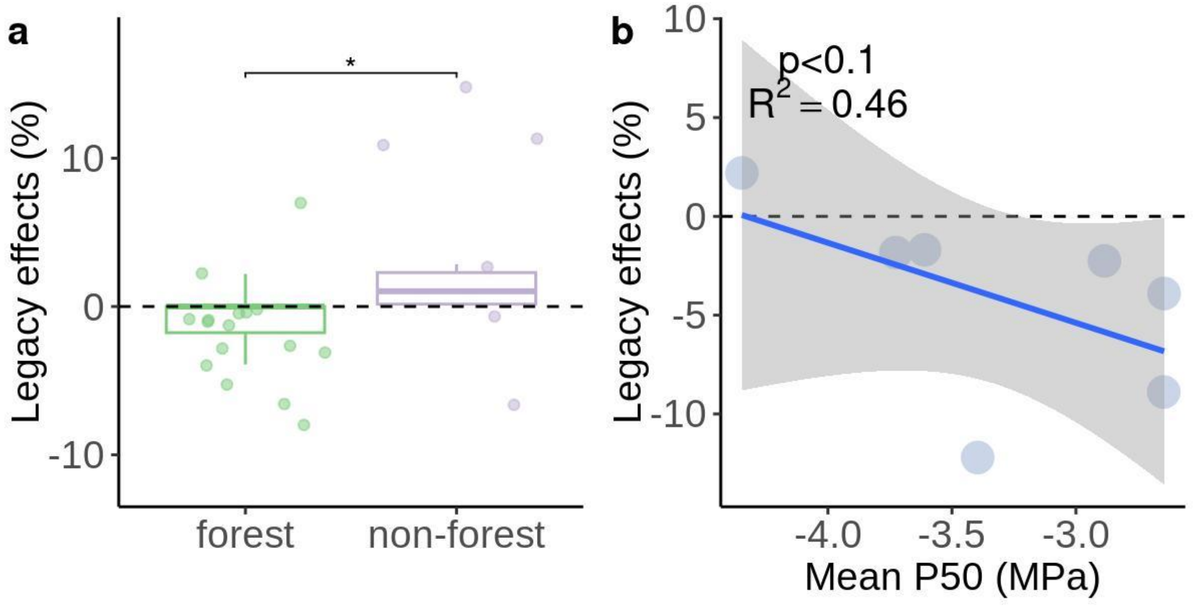

Figure S4. The factors modulating the spatial variability of drought legacy effects on GPP based on the daytime partitioning method. a) Comparison of drought legacy effects on GPP between forests and non-forests. The significance label indicates the difference in legacy effects across forest and non-forest sites is statistically significant (* p< 0.05), based on the independence test (see Methods). **c)** Stronger legacy effects are associated with ecosystems with less negative mean P50 (the water potential at 50% loss of hydraulic conductivity). P50 was upscaled from species weighted by their relative abundance in the footprint of the respective EC tower. The blue line with the grey area indicates the linear regression line and associated uncertainty. Only the drought legacy effects detected are shown here.

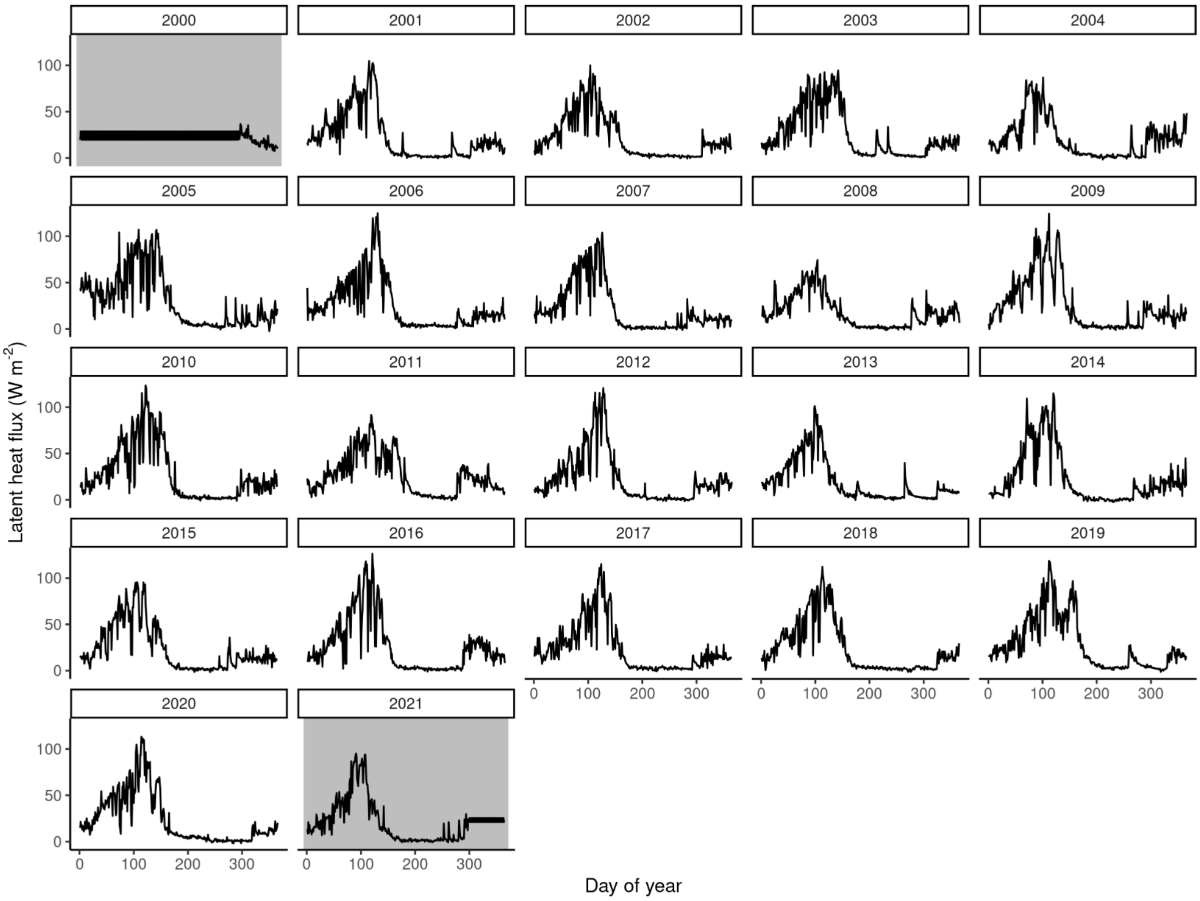

Figure S5. Latent heat flux (LE) at US-Var, a semi-arid grassland in the US. The data in the years 2000 and 2021 were excluded from the analysis due to missing LE data.

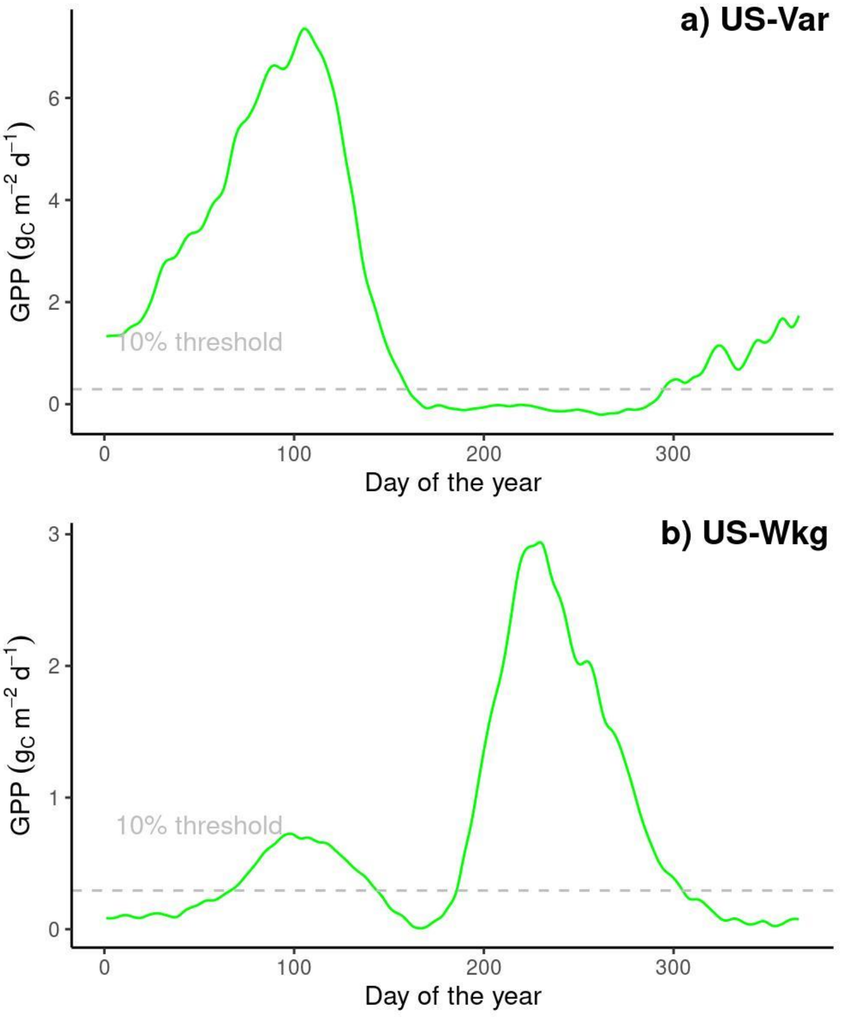

Figure S6. The seasonal cycle of gross primary productivity (GPP) at a) US-Var and b) US-Wkg.

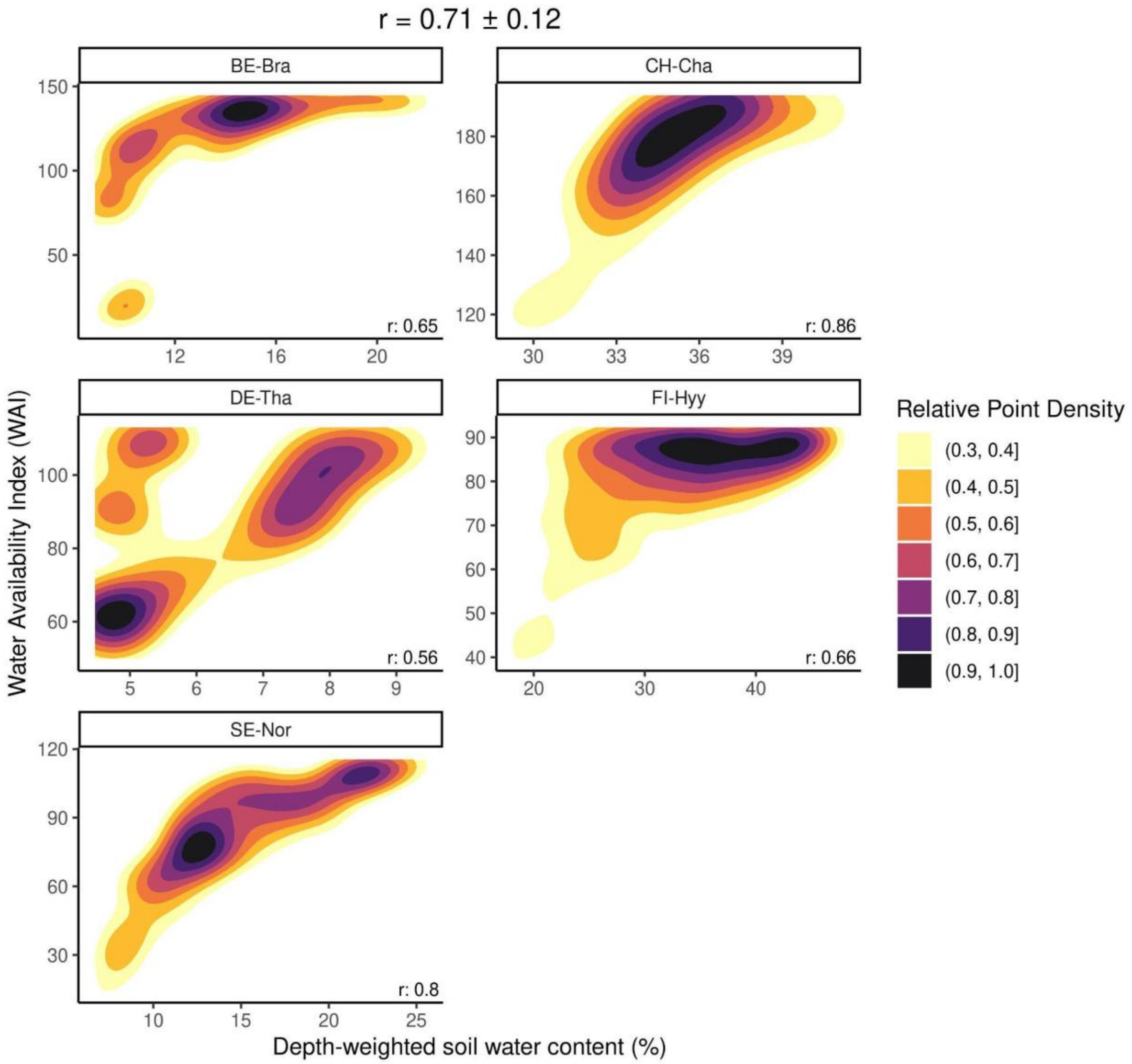

Figure S7. Comparison between depth-weighted soil water content (SWC) and the water availability index (WAI) during the growing seasons across five sites. Depth-weighted SWC is computed by weighting the soil water content from four measurement layers by their corresponding depths, with the deepest layer typically reaching ~50 cm. Only time steps with SWC available at all four layers were included. The correlation coefficient (r) between WAI and depth-weighted SWC is calculated to assess how well WAI captures the observed soil water content dynamics. Colors indicate relative point density, normalized so the highest-density region in each panel equals 1.

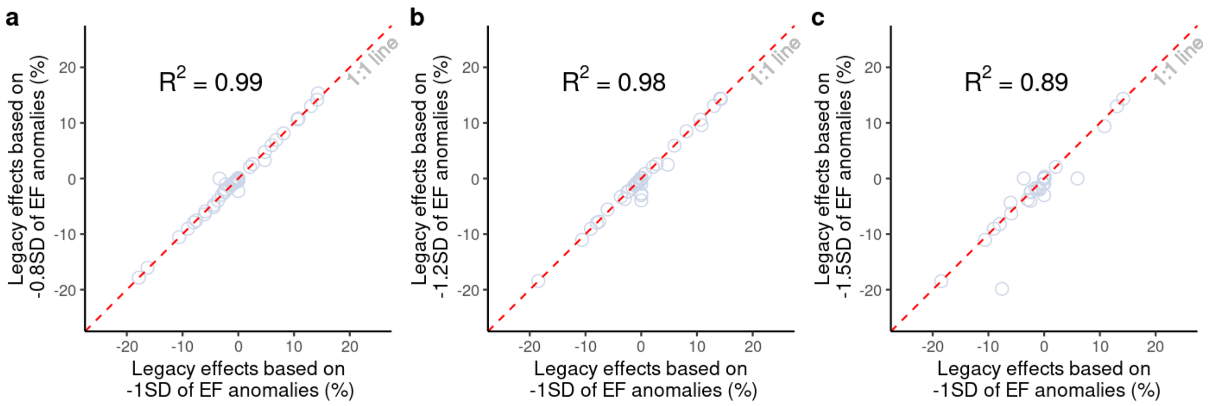

Figure S8. Comparison of legacy effects between drought definitions using different thresholds multiplied by the standard deviation (-0.8SD, -1SD, -1.2SD, and -1.5SD) of evaporative fraction (EF) anomalies. Each point is a drought event. In the Methods, when the daily EF anomaly was lower than the -1 standard deviation of EF anomalies over the entire data record, the time step was flagged as a drought time step.

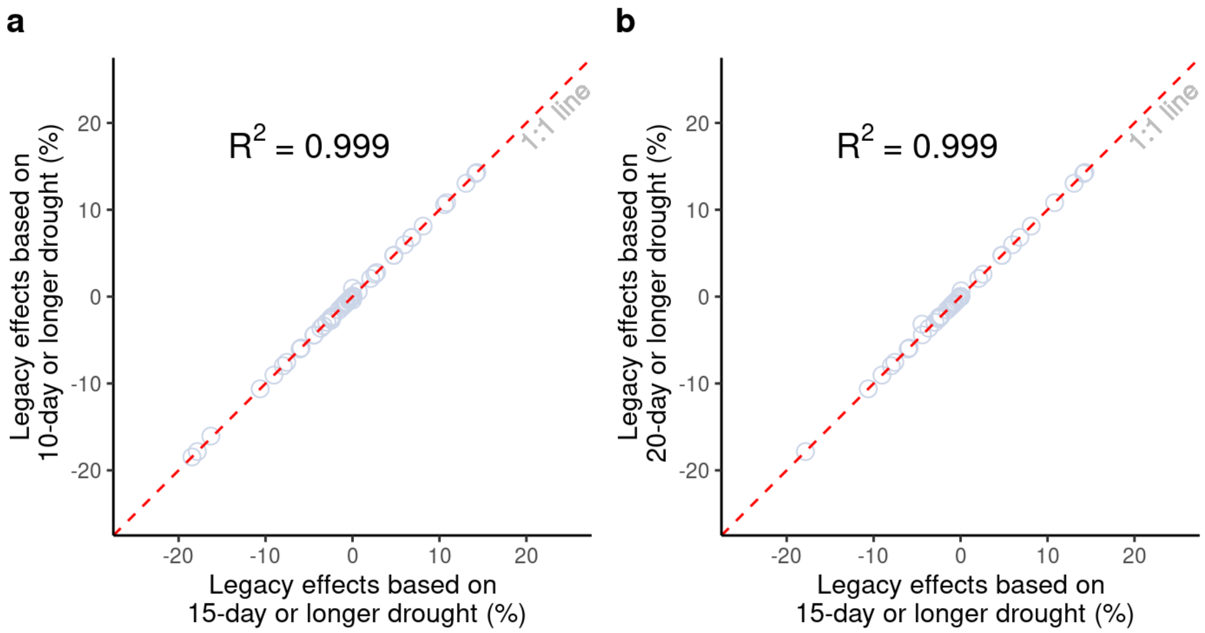

Figure S9. Comparison of legacy effects between drought definitions using different minimum drought lengths (10, 15, and 20 days). Each point is a drought event. In the Methods, we identified a drought event as the period in which there are longer than 15 consecutive drought time steps.

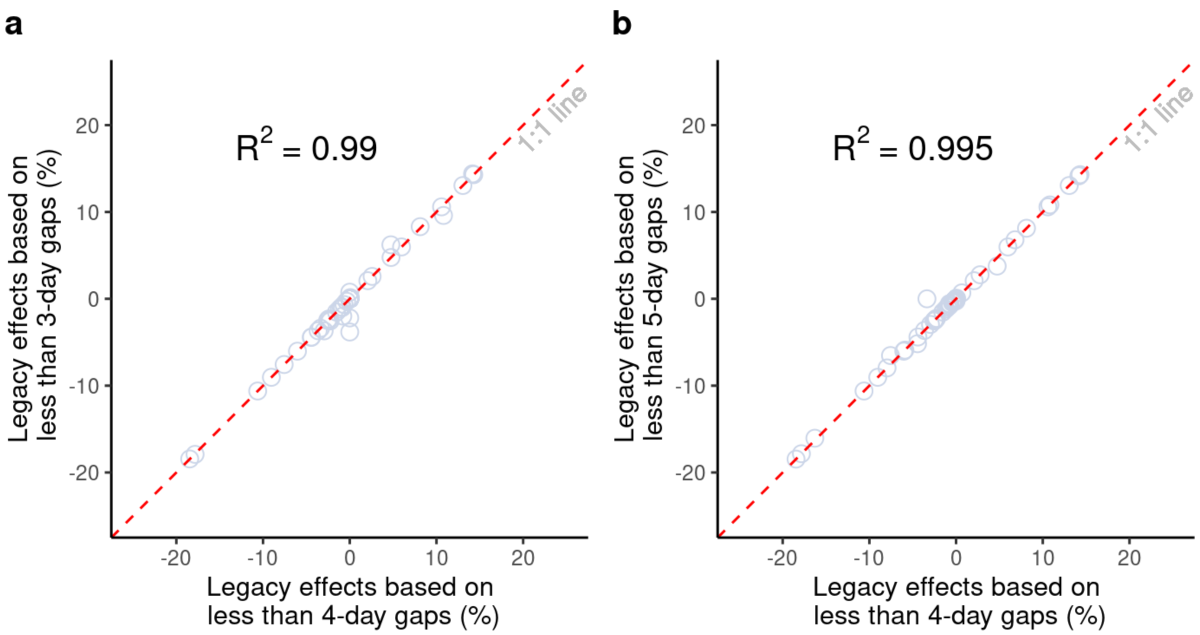

Figure S10. Comparison of legacy effects between drought definitions using different gapped days (less than 3, 4, and 5 days). Each point is a drought event. In the Methods, to avoid the effect of data gaps or noise of the data, if the non-drought time steps between two neighboring drought time steps were less than 4, those non-drought time steps were still flagged as drought time steps.

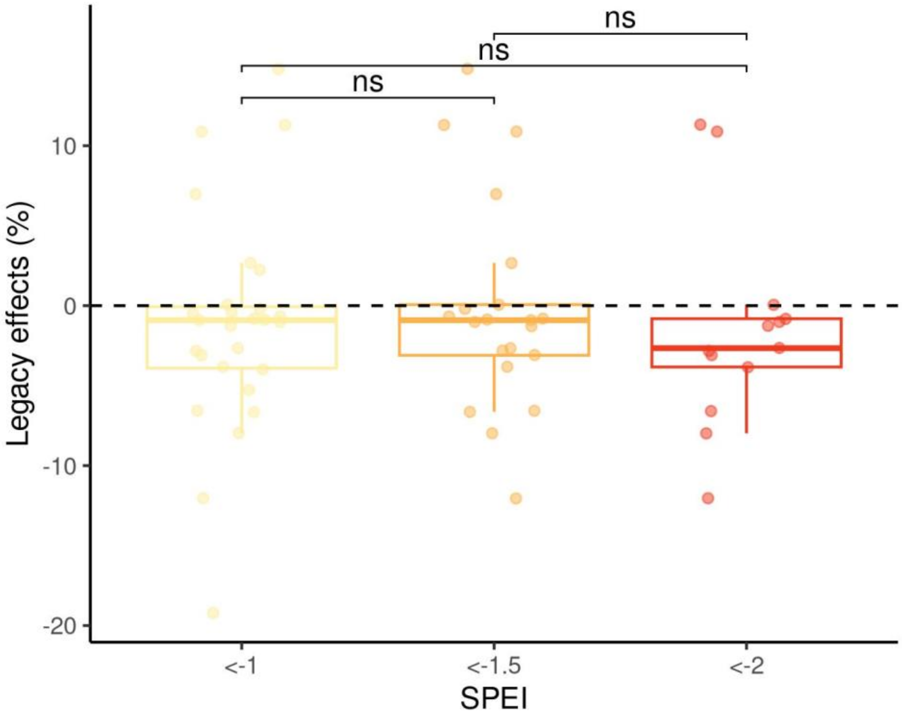

Figure S11. Legacy effects on gross primary productivity along the drought severity classes, i.e., SPEI <-1, <-1.5, and <-2. The significance is tested by a non-paired two-sample Wilcoxon test.

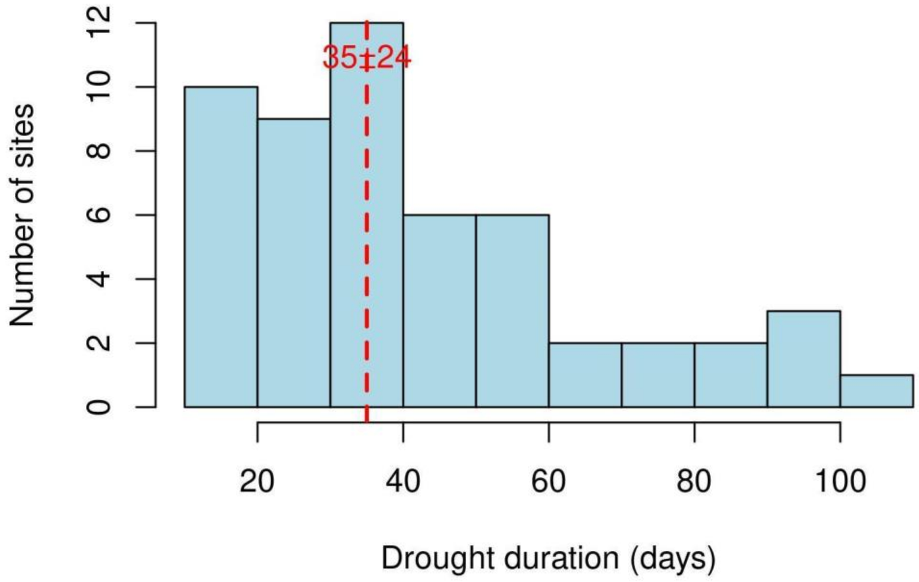

Figure S12. The duration of selected drought events across investigated eddy covariance sites. The dashed line and associated numbers indicate the median value and the standard deviation.

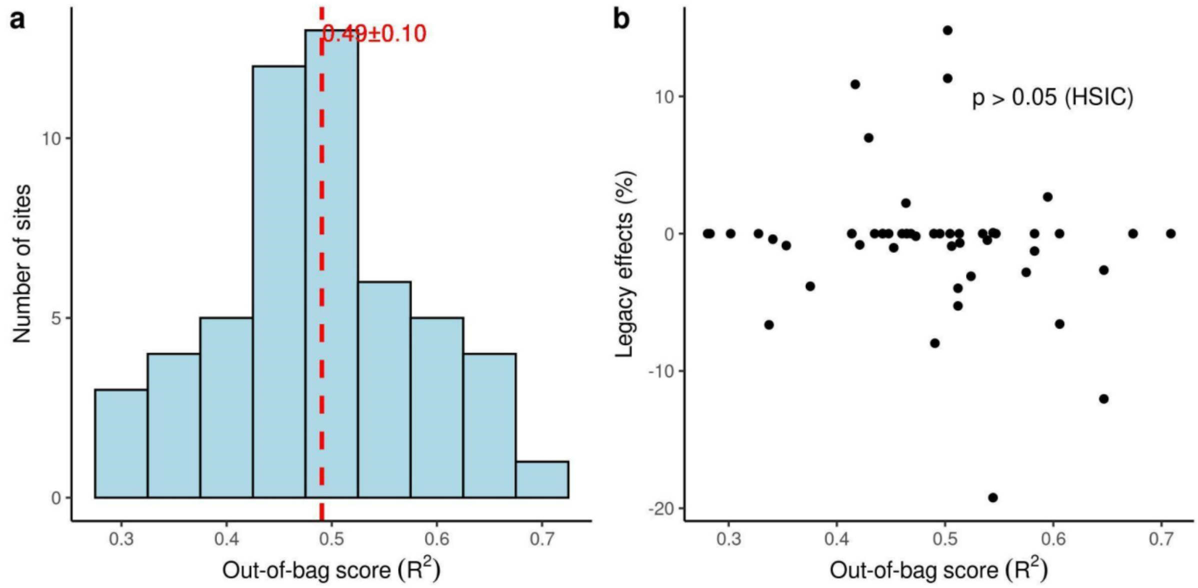

Figure S13. a) The out-of-bag score of trained random forest models across investigated eddy covariance sites and b) its relation to legacy effects. The dashed line and associated numbers in **a)** indicate the median value and the standard deviation. In **b)**, there is no significant relationship between out-of-bag score and legacy effects based on Hilbert-Schmidt independence criterion (HSIC).

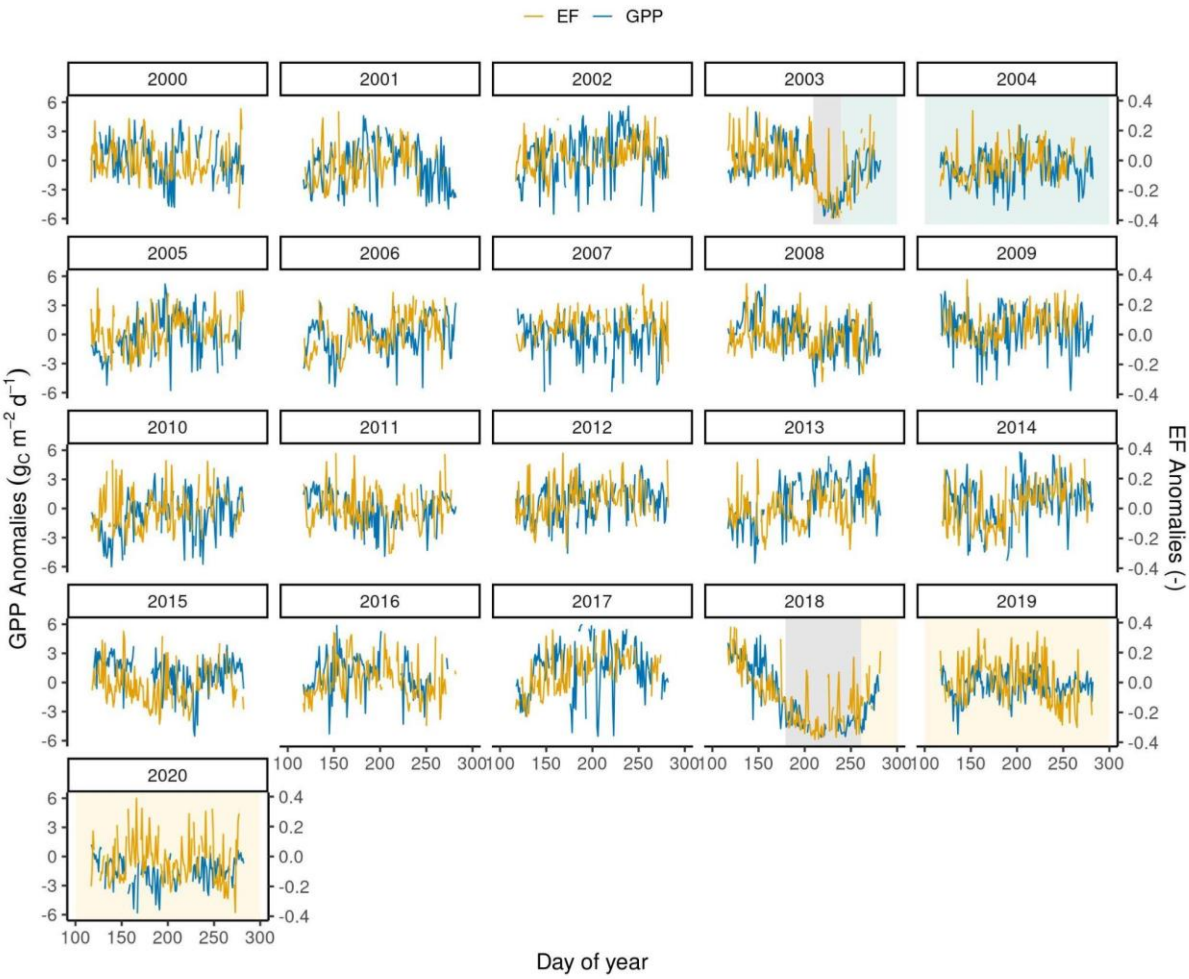

Figure S14. A case example of non-legacy, drought, and legacy periods at DE-Hai, a temperate forest in Germany. Gross primary productivity (GPP) anomalies and evaporative fraction (EF) anomalies time series are in blue and orange colors, respectively. Drought periods are the periods with a grey color. The periods with powder blue and cosmic latte colors are the identified legacy periods of the 2003 and 2018 drought, respectively. Non-legacy periods are the periods with white and grey colors, in which data are used to train the random forest regression model.

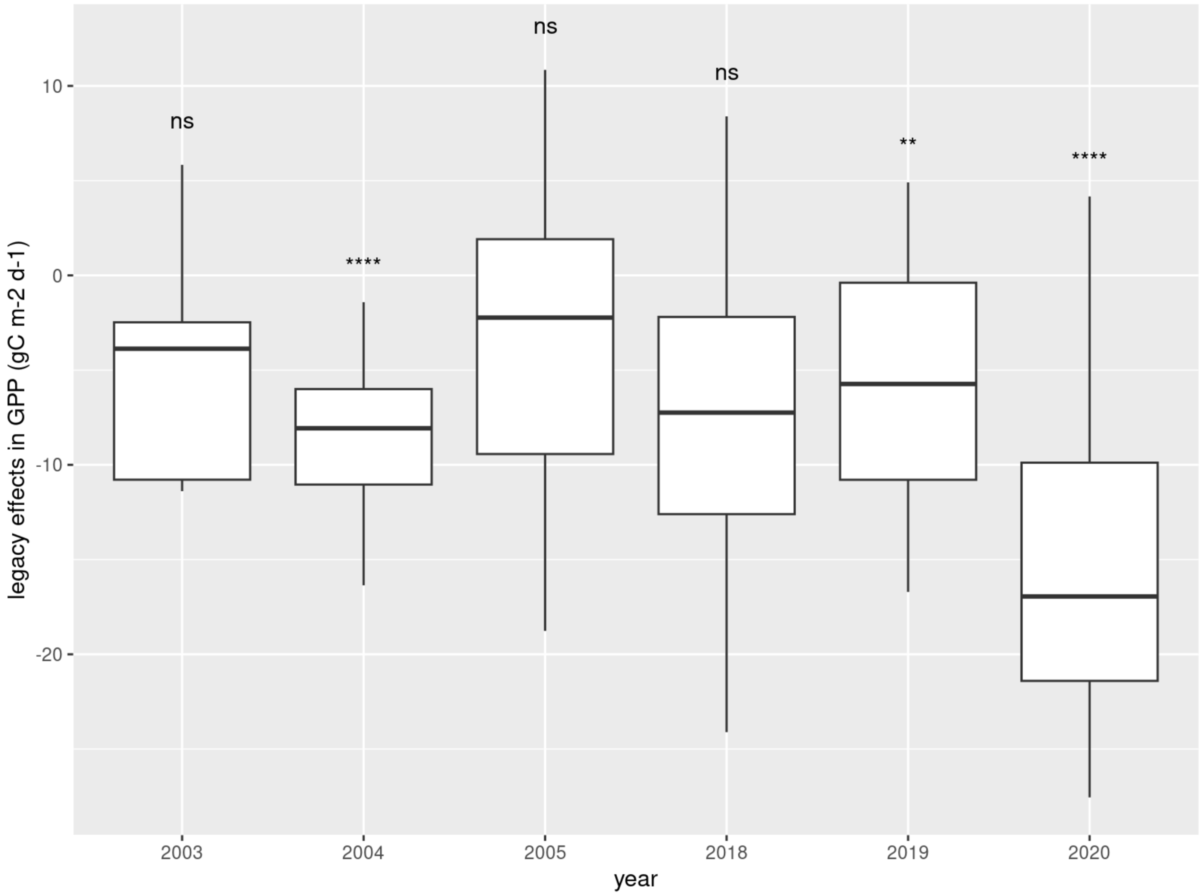

Figure S15. A case example of quantification of the duration of legacy effects at DE-Hai, a temperate forest in Germany. After the 2003 drought, the legacy effects were significantly different from zero in 2004 but non-significant in 2005 (converged), therefore, the duration of legacy effects of the 2003 drought were quantified as the rest of the growing season after the drought ended and the entire 2004 growing season. After the 2018 drought, the legacy effects were significantly different from zero in both the entire growing season of 2019 and 2020, but there is no data record after 2020, therefore the legacy duration of the 2018 drought can not be quantified.

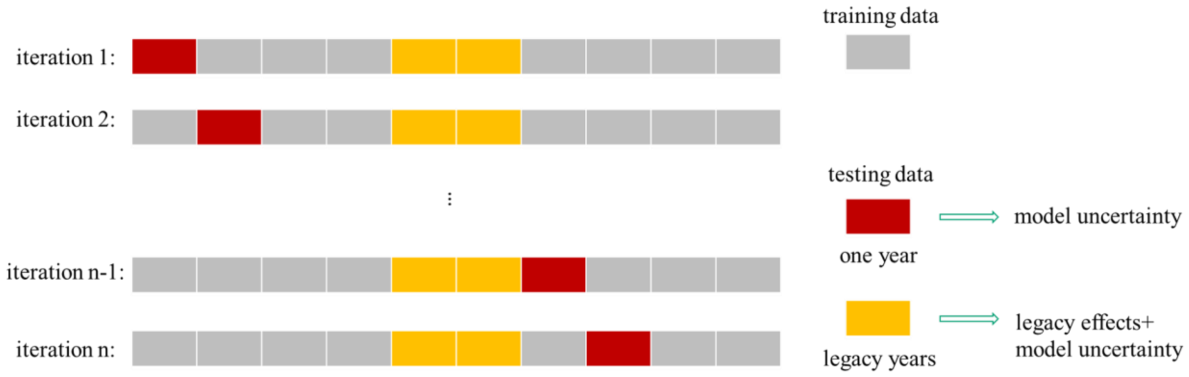

Figure S16. The illustration of the quantification of model uncertainty for each site. Each row is one iteration and n is the number of iterations which is the number of years in the non-legacy periods. Yellow columns indicate the legacy periods, while non-legacy periods include red and grey columns. In each iteration, we only use data of years in grey to train the model and predict in years in both red and yellow. Therefore, the difference between potential and

actual values (residual) in the year in red represents the model uncertainty, while the residual in the year in yellow includes both the legacy effects signal and model uncertainty. After all iterations, we collect daily residuals in years in red and calculate the 5-95% quantiles of the residuals for each day of year. We compare the daily residuals in legacy years with the model uncertainty in the corresponding day of year and only consider when larger than uncertainty.

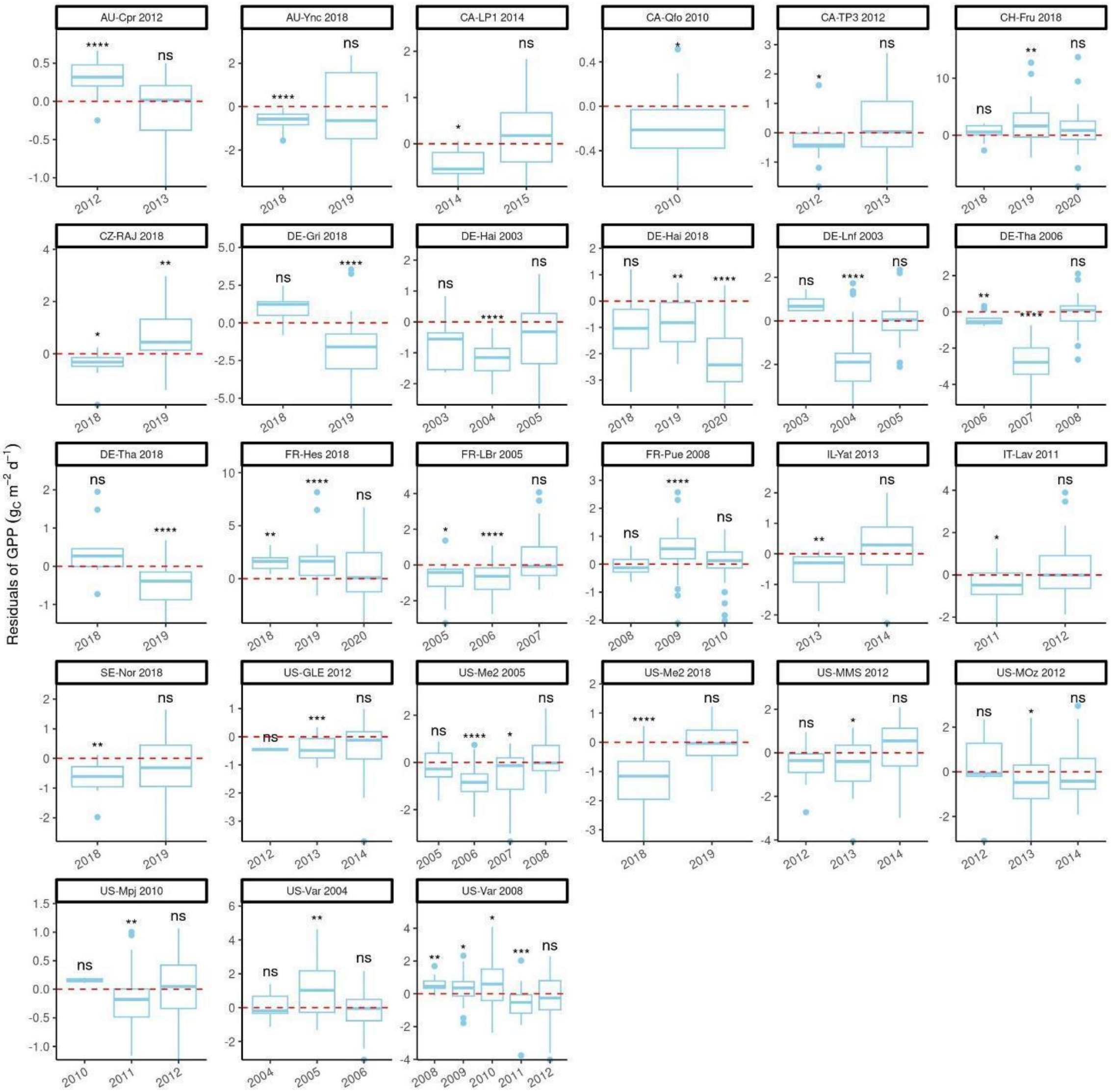

Figure S17. Residuals of GPP during the drought year, legacy years, and one year following the last legacy year for each site. Each subplot title indicates the site ID and the year of the analyzed drought event. Residuals for the remaining growing season after the drought event were also evaluated. If residuals for this period were not significantly different from 0, the subsequent year was analyzed further, as legacy effects may extend beyond the drought year. Significance was assessed using the Wilcoxon test.

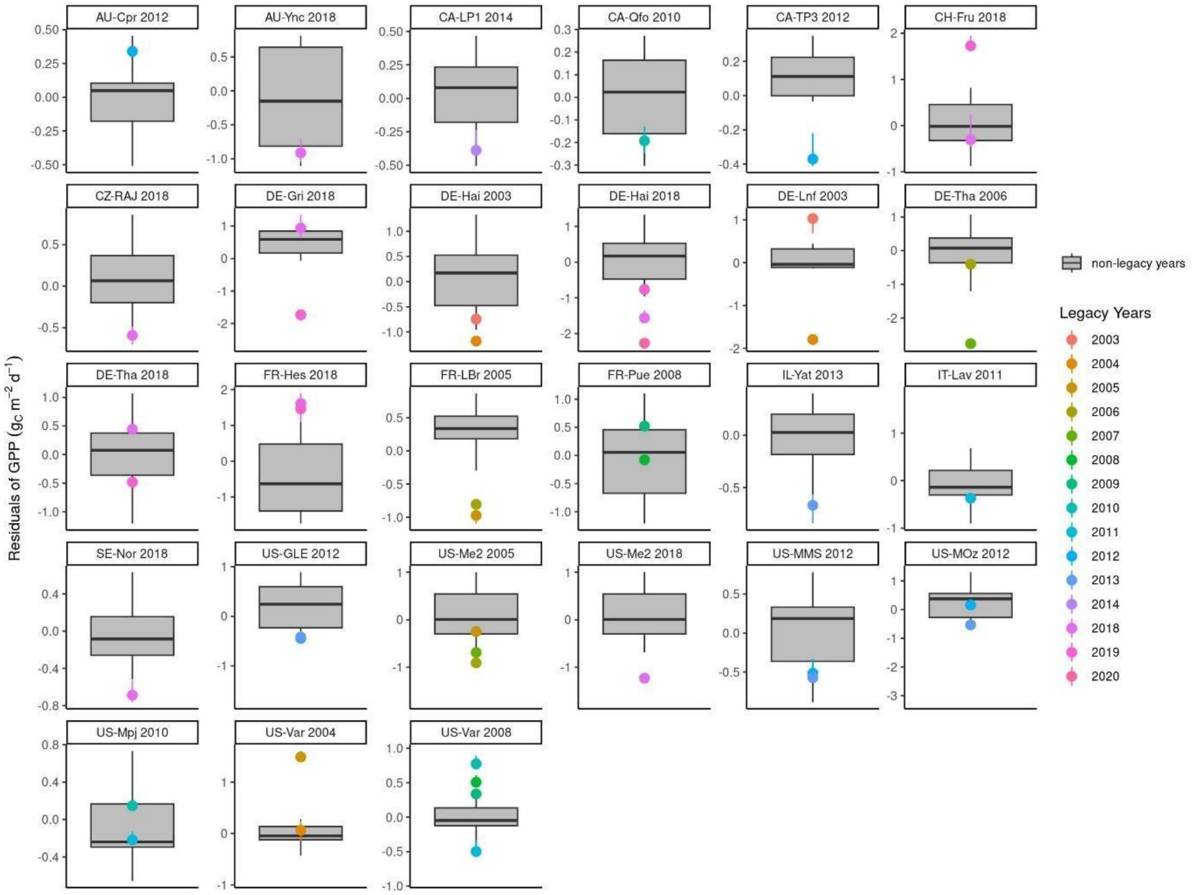

Figure S18. The comparison of residuals of GPP in identified legacy years and the model uncertainty for each site. Each subplot title specifies the site ID and the year of the drought event under investigation.

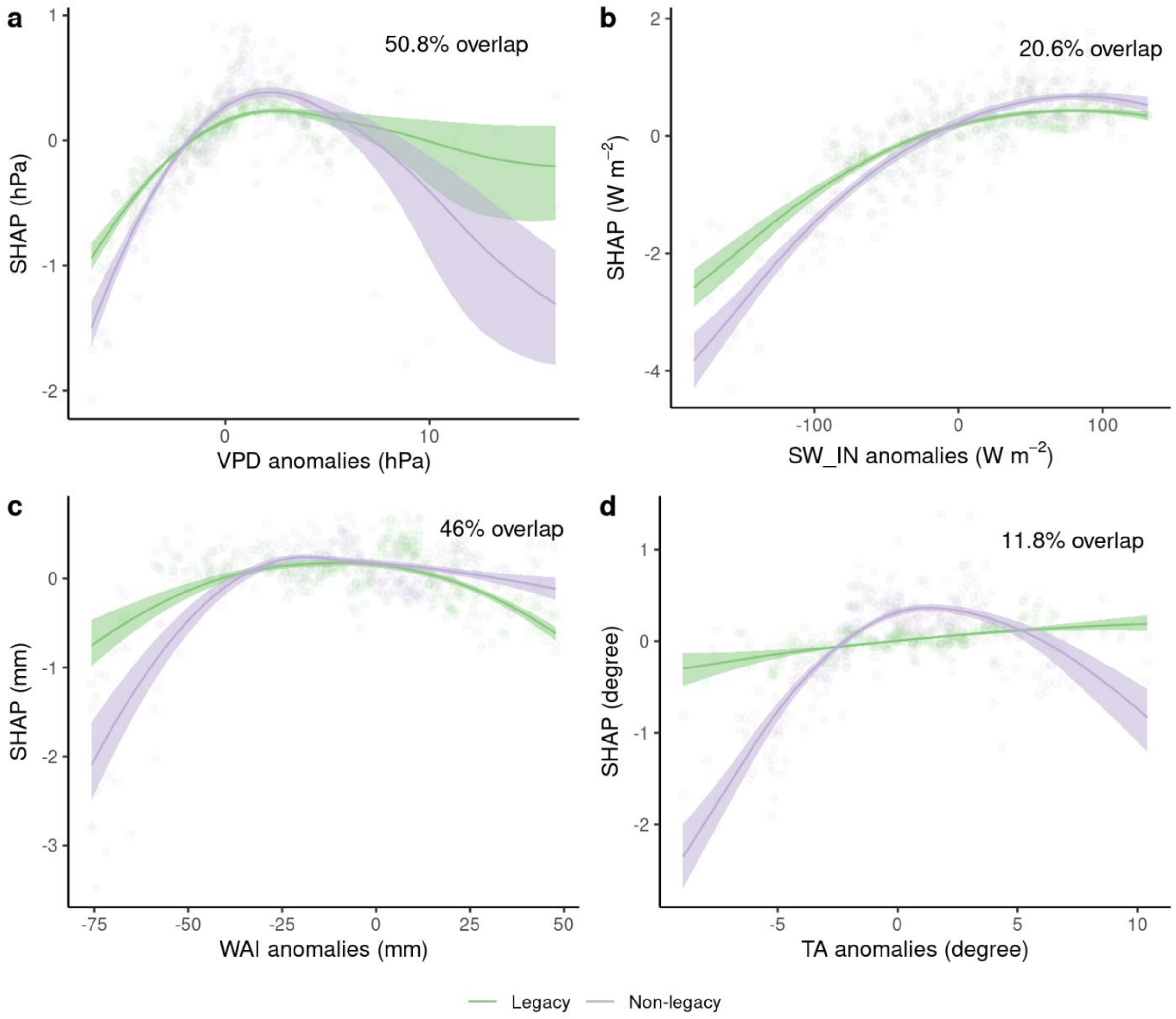

Figure S19. The SHAP responses to a) vapour pressure deficit (VPD), b) incoming shortwave radiation (SW_IN), c) water availability index (WAI), and d) air temperature (TA) anomalies for models trained on legacy periods of the 2018 drought and non-legacy period at DE-Hai, a temperate forest in Germany. Shapley Additive Explanations[1] (SHAP) assigns each observation of each predictor an importance value for the prediction of GPP_{anom}, quantifying how much each observation of each predictor contributes, positively or negatively, to the prediction. To address feature dependence, we applied a conditional SHAP approach that models inter-feature relationships during attribution estimation. To minimize the influence of differing predictor ranges between the legacy and non-legacy periods, we calculated SHAP values only within the common range shared by both periods for each predictor. Additionally, to control for differences in sample size, we subsampled the data in non-legacy periods to match the sample size of legacy periods when training the random forest model. For each predictor, points in green and purple are from legacy periods and non-legacy periods, respectively. The fitted lines are generated by LOESS (locally estimated scatterplot smoothing) with a span of 1. We checked if the 95% confidence interval of the fitted two curves overlapped (see Method). A low overlap percentage indicates a shift in the response of GPP_{anom} to the respective predictor.

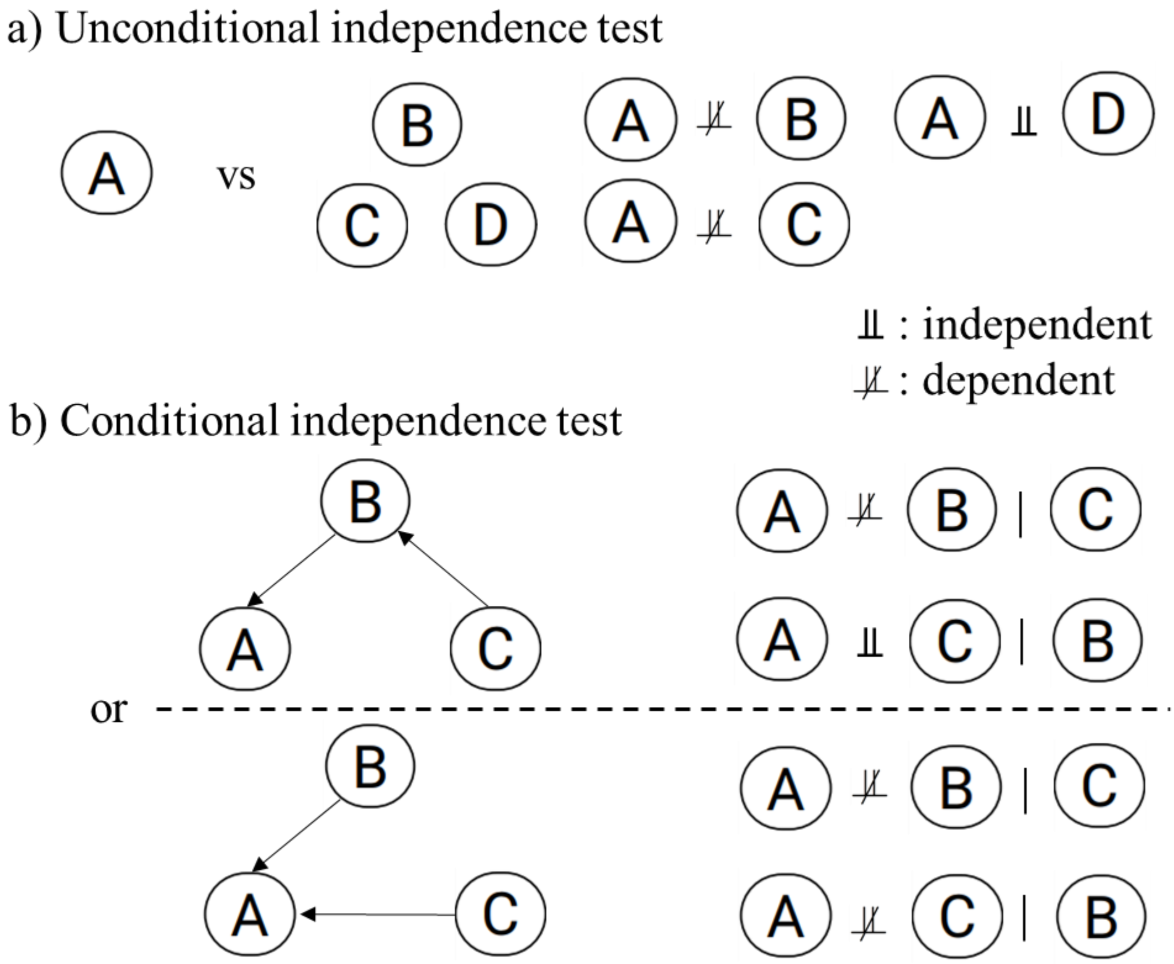

Figure S20. Illustration of a) unconditional independence test and b) conditional independence test. 'A', 'B', 'C', and 'D' circles are variables. "|" is the condition symbol. In the unconditional independence test (a), we find A's dependences on B and C are significant but non-significant on D. However, it is unclear what are the pathways of the influences of B and C on A. There are two possible pathways: 1) C influences A through B (or B influences A through C, which does not show here) or 2) B and C independently influence A. Therefore, we need to do conditional independence test (b) which is to test if A's dependence on B condition on C is still significant or not, and vice versa. If we found A's dependence on B condition on C is still significant while A's dependence on C condition on B is non-significant, we could conclude C influences A through B and B is the causal driver. If we found both A's dependence on B condition on C and A's dependence on C condition on B are still significant, we could conclude B and C both are causal drivers and independently influence A.

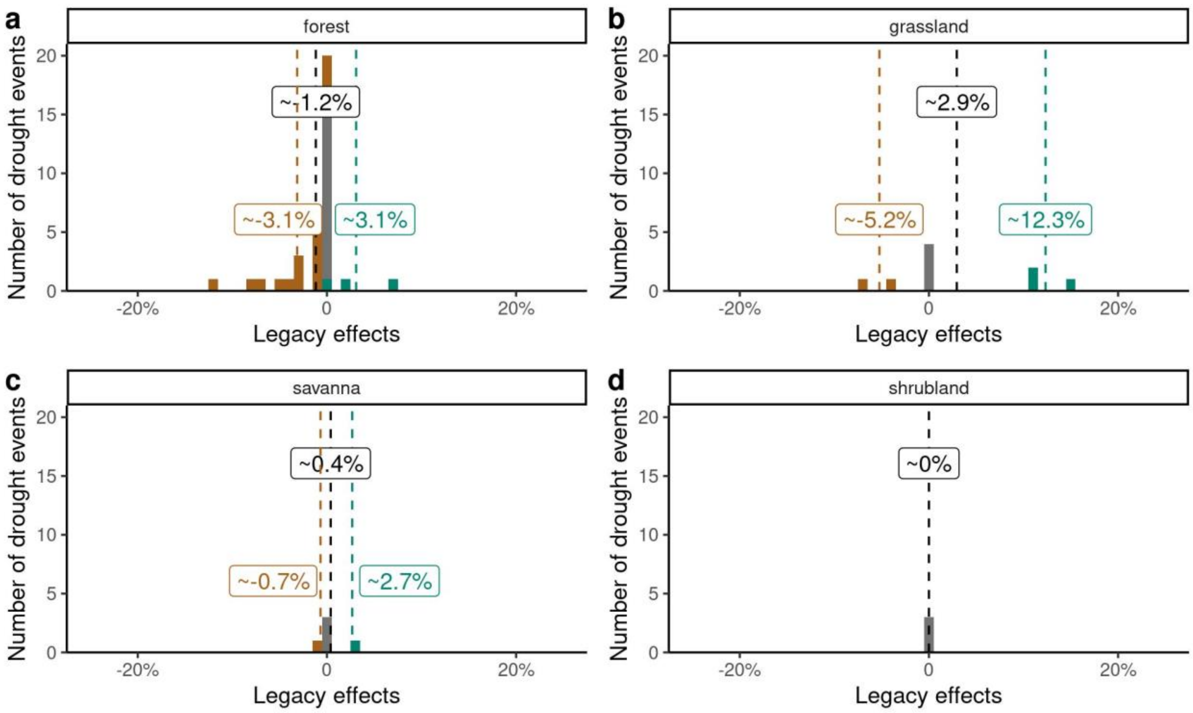

Figure S21. Frequency distribution of positive and negative legacy effects across a) forest, b) grassland, c) savanna, and d) shrubland sites. Values in green and brown are overall average cumulative changes in GPP across sites, relative to the annual mean of GPP, due to positive and negative drought legacy effects, respectively. Values in black are the overall average of both positive and negative drought legacy effects.

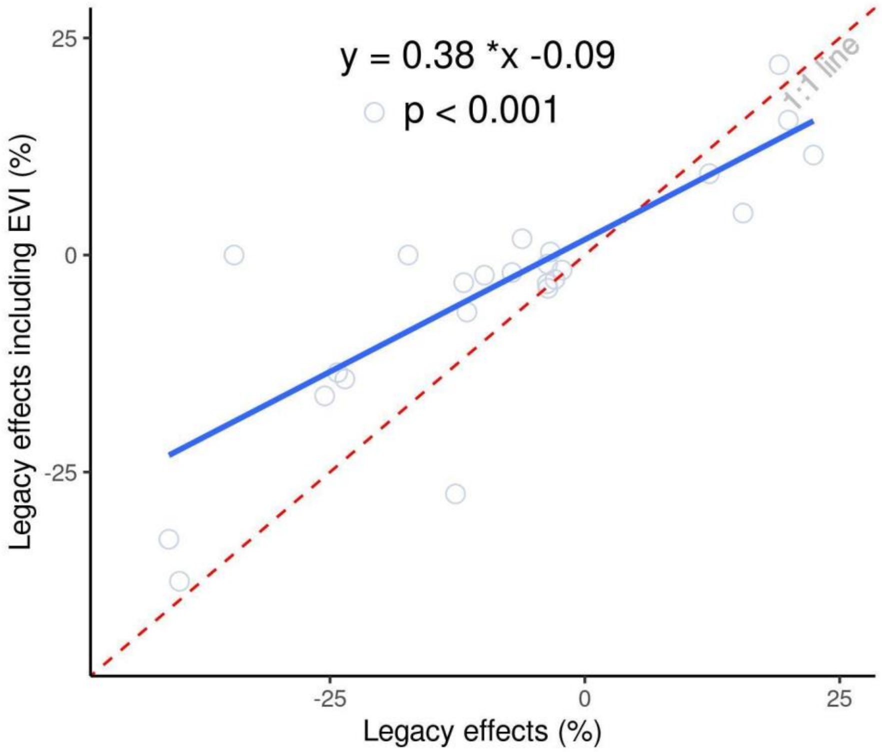

Figure S22. The comparison of legacy effects between using predictors with and without EVI anomalies. The blue line corresponds to the linear regression of legacy effects including uncertainty with EVI as predictor vs. without across all drought events. The slope

indicates the relative effect of adding structural information on the random forest model used to predict potential GPP. A slope smaller than 1 indicates that adding observations of EVI make the model simulate more closely observed GPP, so that part of the departures of observed GPP from potential GPP can be explained by structural canopy changes.

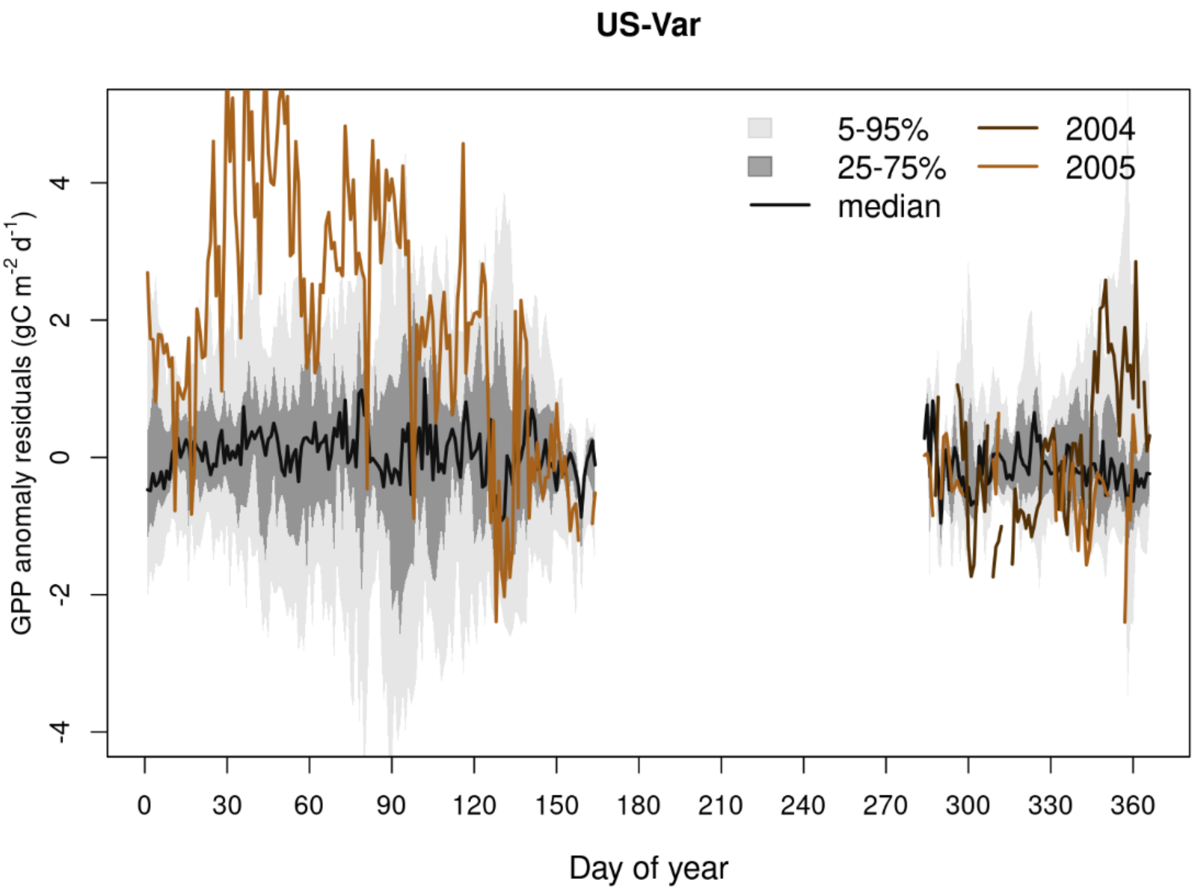

Figure S23. Legacy effects on GPP of the 2004 drought at the seasonal scale at US-Var, a semi-arid grassland in the US. The colored lines show the GPP_{anom} residuals in the legacy periods which include the end of the growing season of 2004 and the entire growing season of 2005 and the drought period is not shown here. The black line and shaded areas are the model uncertainties characterized by the median, the 25th–75th, and 5th–95th quantile ranges of GPP_{anom} residuals in non-legacy years. Negative residuals corresponded to more negative or less positive GPP_{anom} than would be expected given the climate in that year, indicating negative legacies of drought, while positive residuals corresponded to less negative or more positive GPP_{anom}, indicating beneficial legacies of drought.

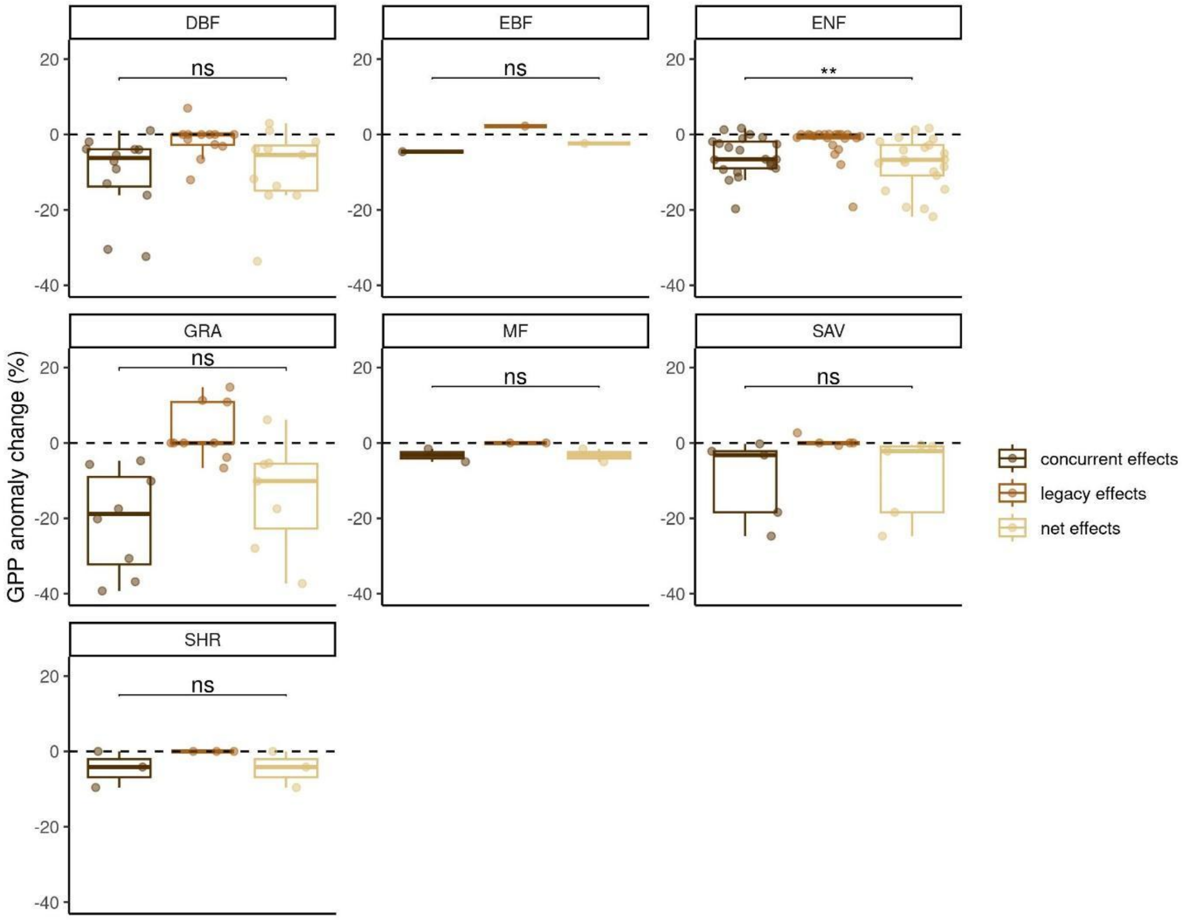

Figure S24. The concurrent, legacy, and net effects of drought on GPP across biomes. Net effects are the sum of concurrent and legacy effects of drought events. Each point indicates the percent change in cumulative GPP anomalies induced by concurrent, legacy, or net effects of drought relative to the long-term mean based on the entire record at the respective site. The significance labels indicate whether the difference between concurrent and net effects is statistically significant (**, $p<0.05$) or not (ns, $p<0.05$), based on a paired two-sample Wilcoxon test. Each panel is for each plant functional type: DBF, deciduous broad-leaf forests; EBF, evergreen broad-leaf forests; ENF, Evergreen needle-leaf forests; GRA, grassland; MF, mixed forests; SAV, savanna and woody savanna; SHR, closed shrublands and open shrublands.

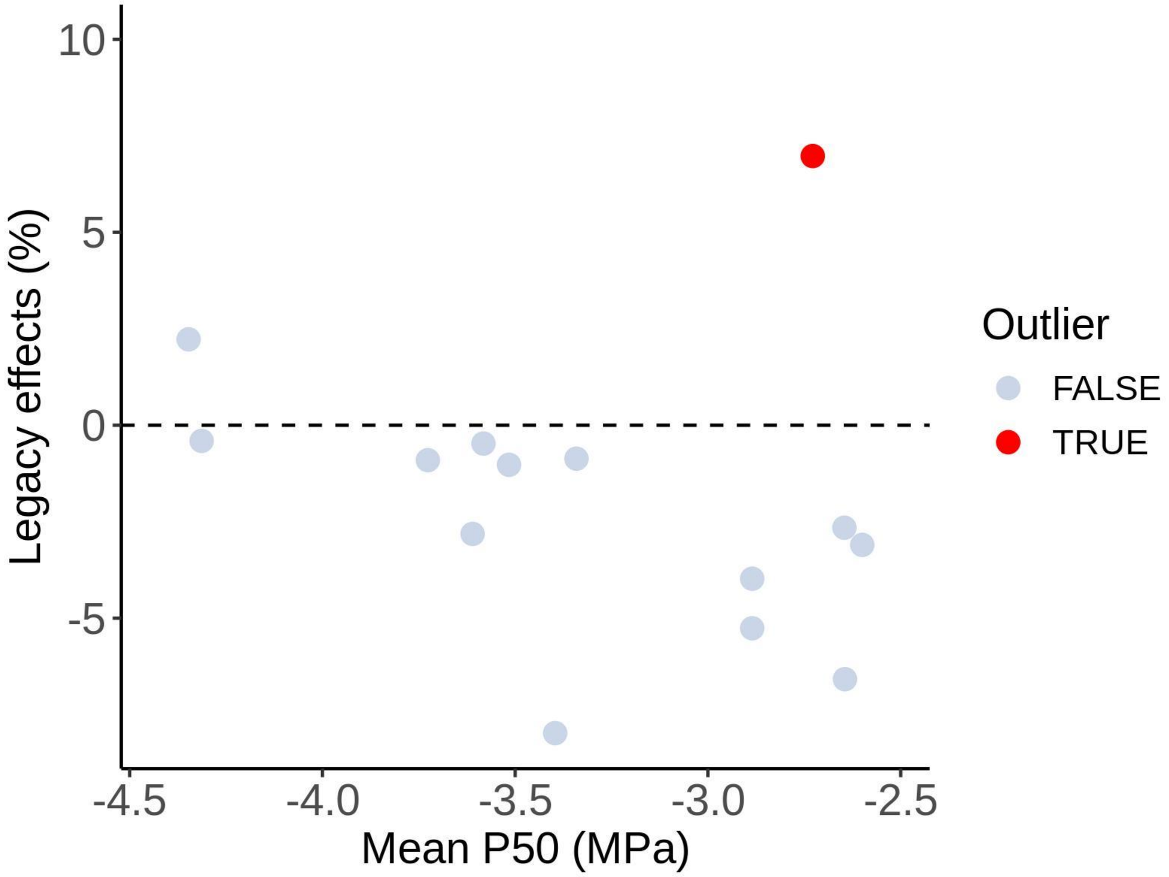

Figure S25: Relationship between mean P50 (MPa) and legacy effects (%). An outlier (in red) was identified using Mahalanobis distance, which accounts for the multivariate distribution of the data. Most points (in grey) fall within the expected multivariate range, while the outlier deviates significantly from the joint distribution of the variables.
