# Supplementary material for: Widespread but Divergent Drought Legacy Effects on Gross Primary Productivity Across Biomes
Source: Glob Chang Biol. 2025 Oct 10;31(10):e70541. doi: 10.1111/gcb.70541 (PMC12514344; doi:10.1111/gcb.70541)
Supplement: Supplementary file 2 — Tables S1–S7. gcb70541‐sup‐0002‐TableS1‐S7.pdf. [file GCB-31-e70541-s001.pdf]

Contents in this file include:
Supplementary Tables 1-7

Table S1. List of investigated eddy covariance sites. Plant functional types (PFT): DBF, deciduous broad-leaf forests; EBF, evergreen broad-leaf forests; ENF, Evergreen needle-leaf forests; GRA, grassland; MF, mixed forests; SAV, savanna; WSA: woody savanna; CSH, closed shrublands; and OSH, open shrublands.

site	PFT	Dataset	Investigated years	data citation
AT-Neu	GRA	FLUXNET 2015	2002-2012	Wohlfahrt et al. (2016)
AU-ASM	SAV	OzFlux	2010-2021	Cleverly et al. (2021)
AU-Cpr	SAV	OzFlux	2010-2021	Tech (2013)
AU-Ctr	EBF	OzFlux	2010-2018	Liddell (2013a)
AU-DaS	SAV	OzFlux	2008-2021	Beringer (2013a)
AU-Gin	WSA	OzFlux	2011-2020	Silberstein (2015)
AU-GWW	SAV	OzFlux	2013-2021	Macfarlane (2013)
AU-How	WSA	OzFlux	2002-2021	Beringer (2013b)
AU-Rob	EBF	OzFlux	2013-2021	Liddell (2013b)
AU-Stp	GRA	OzFlux	2008-2021	Beringer (2013c)
AU-Tum	EBF	OzFlux	2002-2011	Woodgate (2013)
AU-Wom	EBF	OzFlux	2010-2020	Arndt (2013)
AU-Ync	GRA	OzFlux	2013-2021	Beringer (2013d)
BE-Bra	MF	ICOS Warm Winter 2020	2004-2019	Janssens et al. (2022)

BE-Vie	MF	ICOS Warm Winter 2020	2010-2020	Vincke et al. (2022)
CA-Gro	MF	AmeriFlux FLXUNET	2004-2013	McCaughey (2022)
CA-LP1	ENF	AmeriFlux FLUXNET	2007-2016	Black (2021)
CA-Obs	ENF	AmeriFlux FLUXNET	2000-2010	Black (2018)
CA-Qfo	ENF	AmeriFlux FLUXNET	2004-2010	Margolis (2023)
CA-TP1	ENF	FLUXNET 2015	2008-2014	Arain (2016a)
CA-TP3	ENF	FLUXNET 2015	2008-2014	Arain (2016b)
CH-Cha	GRA	ICOS Warm Winter 2020	2010-2020	Buchmann, Merbold, et al. (2022)
CH-Dav	ENF	ICOS Warm Winter 2020	1997-2019	Buchmann, Hörtnagl, et al. (2022)
CH-Fru	GRA	ICOS Warm Winter 2020	2010-2020	Buchmann, Eugster, Hörtnagl, et al. (2022)
CH-Lae	MF	ICOS Warm Winter 2020	2005-2019	Buchmann, Eugster, Paul-Limoges, et al. (2022)
CH-Oe1	GRA	FLUXNET 2015	2002-2008	Ammann (2016)
CZ-BK1	ENF	ICOS Warm Winter 2020	2004-2019	Sigut, Foltýnová, Kowalska, et al. (2022)
CZ-RAJ	ENF	ICOS Warm Winter 2020	2012-2019	Sigut, Foltýnová, Czerný, et al. (2022a)
CZ-Stn	DBF	ICOS Warm Winter 2020	2010-2019	Sigut, Foltýnová, Czerný, et al. (2022b)

DE-Gri	GRA	ICOS Warm Winter 2020	2004-2019	Bernhofer et al. (2022a)
DE-Hai	DBF	ICOS Warm Winter 2020	2000-2020	Knohl et al. (2022)
DE-Lnf	DBF	FLUXNET 2015	2002-2006, 2010-2012	Knohl et al. (2016)
DE-RuR	GRA	ICOS Warm Winter 2020	2012-2019	Schmidt, Graf, Dolfus, et al. (2022)
DE-RuW	ENF	ICOS Warm Winter 2020	2012-2019	Schmidt, Graf, Drüe, et al. (2022)
DE-Tha	ENF	ICOS Warm Winter 2020	1997-2019	Bernhofer et al. (2022b)
DK-Sor	DBF	FLUXNET 2015	1997-2013	Ibrom & Pilegaard (2016)
ES-Agu	OSH	ICOS Warm Winter 2020	2007-2014	Poveda et al. (2022)
ES-LJu	OSH	FLUXNET 2015	2005-2011	Cañete et al. (2016)
FI-Hyy	ENF	ICOS Warm Winter 2020	1996-2019	Mammarella et al. (2022)
FI-Sod	ENF	FLUXNET 2015	2008-2014	Aurela et al. (2016)
FR-FBn	MF	ICOS Warm Winter 2020	2008-2019	Simioni et al. (2022)
FR-Fon	DBF	ICOS Warm Winter 2020	2006-2020	Dufrêne et al. (2022)
FR-Hes	DBF	ICOS Warm Winter 2020	2014-2020	Cuntz et al. (2022)
FR-LBr	ENF	FLUXNET 2015	2001-2008	Berbigier & Loustau (2016)

FR-Pue	EBF	FLUXNET 2015	2001-2014	Ourcival (2016)
IL-Yat	ENF	ICOS Warm Winter 2020	2012-2020	Yakir et al. (2022)
IT-Cpz	EBF	FLUXNET 2015	2001-2008	Valentini et al. (2016)
IT-Lav	ENF	ICOS Warm Winter 2020	2003-2019	Gianelle et al. (2022)
IT-Noe	CSH	FLUXNET 2015	2005-2014	Spano et al. (2016)
IT-Ren	ENF	ICOS Warm Winter 2020	1999-2020	Montagnani et al. (2022)
IT-Ro2	DBF	FLUXNET 2015	2002-2008	Papale et al. (2016)
IT-SR2	ENF	ICOS Warm Winter 2020	2013-2019	Arriga et al. (2022)
IT-SRo	ENF	FLUXNET 2015	2004-2010	Gruening al. (2016)
IT-Tor	GRA	ICOS Warm Winter 2020	2009-2019	Cremonese et al. (2022)
NL-Loo	ENF	FLUXNET 2015	1997-2014	Moors & Elbers (2016)
RU-Fyo	ENF	ICOS Warm Winter 2020	1999-2019	Varlagin et al. (2022)
SE-Nor	ENF	ICOS Warm Winter 2020	2014-2020	Mölder et al. (2022)
US-Bar	DBF	AmeriFlux FLUXNET	2005-2017	Richardson & Hollinger (2023)
US-Blo	ENF	FLUXNET 2015	2000-2006	Goldstein (2016)
US-GLE	ENF	AmeriFlux FLUXNET	2005-2020	Massman (2022)

US-Ha1	DBF	AmeriFlux FLUXNET	1992-2020	Munger (2022)
US-Ho2	ENF	AmeriFlux FLUXNET	2000-2018	Hollinger (2022)
US-IB2	GRA	FLUXNET2015	2005-2011	Matamala (2016)
US-Me2	ENF	AmeriFlux FLUXNET	2002-2020	Law (2022)
US-MMS	DBF	AmeriFlux FLUXNET	1999-2020	Novick & Phillips (2022)
US-MOz	DBF	AmeriFlux FLUXNET	2005-2019	Wood & Gu (2022)
US-Mpj	WSA	AmeriFlux FLUXNET	2008-2020	Litvak (2021)
US-NR1	ENF	AmeriFlux FLUXNET	2007-2016	Blanken et al. (2022)
US-PFa	MF	FLUXNET 2015	1997-2014	Desai et al. (2016)
US-Seg	GRA	AmeriFlux FLUXNET	2007-2017	Litvak (2023a)
US-Ses	OSH	AmeriFlux FLUXNET	2007-2017	Litvak (2023b)
US-Ton	WSA	FLUXNET 2015	2002-2014	Baldocchi & Ma (2016)
US-Var	GRA	AmeriFlux FLUXNET	2001-2020	Ma et al. (2023)
US-Whs	OSH	AmeriFlux FLUXNET	2008-2020	Scott (2023a)
US-Wjs	SAV	AmeriFlux FLUXNET	2008-2020	Litvak (2022)

US-Wkg	GRA	AmeriFlux FLUXNET	2005-2020	Scott (2023b)

Table S2. The shift of climatic dependence of GPP_{anom} between non-legacy periods and legacy periods in the drought events which lead to significant legacy effects. We separately trained two random forest models for non-legacy and legacy periods for each site in which we detected significant legacy effects. We then evaluated the contribution of each observation of each predictor to the prediction of GPP_{anom}, by calculating Shapley Additive Explanations (SHAP, Lundberg and Lee, 2017). Based on the Shapley values from cooperative game theory (Shapley, 1953), SHAP assigns each observation of each predictor an importance value for the prediction of GPP_{anom}, quantifying how much each observation of each predictor contributes, positively or negatively, to the prediction. For each predictor, we fitted two response curves of SHAP to the predictor for legacy and non-legacy periods separately based on LOESS (locally estimated scatterplot smoothing) with a span of 1. We checked how much the 95% confidence interval of the fitted two curves overlapped (Figure S1). The values in the table are the overlap percentages. A low overlap percentage indicates a shift in the response of GPP_{anom} to the respective predictor. SW_IN, TA, VPD, and WAI are incoming short-wave radiation, air temperature, vapour pressure deficit, and water availability index. Plant functional types (PFT): DBF, deciduous broad-leaf forests; EBF, evergreen broad-leaf forests; ENF, Evergreen needle-leaf forests; GRA, grassland; MF, mixed forests; SAV, savanna; WSA: woody savanna; CSH, closed shrublands; and OSH, open shrublands.

site	PFT	Drought year	Legacy duration (yr)	SW_IN overlap percentage (%)	TA overlap percentage (%)	VPD overlap percentage (%)	WAI overlap percentage (%)
AU-Cpr	SAV	2012	0	100	25.4	32.4	55.5
AU-Ync	GRA	2018	0	59.5	36.7	65	73.8
CA-LP1	ENF	2014	0	69.6	100	79.8	90.1
CA-Qfo	ENF	2010	0	100	60.4	100	100
CA-TP3	ENF	2012	0	25.2	75.3	57	54.4
CH-Fru	GRA	2018	1	44.6	64.4	20.8	19.3
CZ-RAJ	ENF	2018	0	27.5	36.4	28.6	47.9
DE-Gri	GRA	2018	1	15.6	79.9	83.5	5.8
DE-Hai	DBF	2003	1	37.5	72.3	58	86.5
DE-Hai	DBF	2018	2	20.5	11.9	50.1	45.6

DE-Lnf	DBF	2003	1	79.2	18.2	70.5	21.3
DE-Tha	ENF	2018	1	88.6	87.1	79.5	82
FR-Hes	DBF	2018	1	90.8	100	47.7	44.4
FR-LBr	ENF	2005	1	28.8	35.1	35	90.1
FR-Pue	EBF	2008	1	88.1	21.9	90.2	62.7
IL-Yat	ENF	2013	0	50.2	44.5	57.5	82
IT-Lav	ENF	2011	0	100	76.6	90.5	100
SE-Nor	ENF	2018	0	28.5	34.2	35.1	37.7
US-GLE	ENF	2012	1	60.9	67.1	100	96
US-Me2	ENF	2005	2	59.8	7.9	66.5	82.5
US-Me2	ENF	2018	0	24	23.9	9	11.2
US-MMS	DBF	2012	1	100	92.9	79.8	44.9
US-MOz	DBF	2012	1	46.7	64.2	48.5	78.1
US-Mpj	WSA	2010	1	50.9	42.8	18.7	14.5
US-Var	GRA	2004	1	66.2	36.7	42.5	9.8
US-Var	GRA	2008	3	29.1	100	38.2	22.9

Table S3. The definition of potential drivers to explain the spatial variability of legacy effects and the motivation for the selection of these drivers.

Categories	motivation	Potential drivers	definition
		Forest vs non-forest	Forests include deciduous broad-leaf forests, evergreen broad-leaf forests, evergreen needle-leaf forests and mixed forests, while non-forests are grasslands, savannas, woody savannas, closed shrublands, and open shrublands.
impact index	Drought resulting in more concurrent impact during drought was expected to cause stronger legacy effects	Concurrent impact peak	the 5th quantile of GPP_{anom} during drought periods normalized relative to long-term daily mean GPP; the 5th quantile is used instead of the minumim to reduce the influence of outliers
		Concurrent effects	the sum of GPP_{anom} during drought periods normalized relative to long-term growing-season mean GPP

Drought characteristics	Drought characteristics including its intensity, timing, and duration could influence the drought impact on the ecosystem[16]	Drought intensity	the 5th quantile of WAI anomalies in the drought period, normalized relative to the overall standard deviation; the 5th quantile is used instead of the minumim to reduce the influence of outliers
		Drought duration	the length of the drought period normalized relative to the growing season length
		Drought timing	the onset of the drought period relative to the growing season
Pre-drought and post-drought water limitation	The pre-drought and post-drought water limitation may alter the drought legacy effects.	Pre-drought water limitation	the sum of negative EF_{anom} in the one year before drought normalized relative to the overall standard deviation at the respective site
		Post-drought water limitation	the sum of negative EF_{anom} in the duration of legacy effects normalized relative to the overall standard deviation at the respective site
Background climate condition	Background climate condition served as an evolutionary selector to shape ecosystem composition and vegetation traits associated with drought resistance/resilience.	Aridity	the ratio between mean annual precipitation over mean annual net radiation averaged over the temporal coverage of entire years for each site
		Mean temperature (TA (mean))	the mean annual temperature over the studied period of each site
		Precipitation seasonality (P (CV))	the coefficient of variation (CV) of daily precipitation over the studied period of each site
		Temperature seasonality (TA (CV))	the coefficient of variation (CV) of daily temperature over the studied period of each site
Forest-specific			
site metrics (Table S6)	Site metrics, including forest age, species richness, and rooting depth, were generally relevant to drought resistance/resilience.	forest mean age (age (mean))	the time since management-induced disturbance is normalized to the year 2017, which was collected from the literature, the BADM product, and or site principal investigators (Besnard et al. 2018; Musavi et al. 2017)
		maximum canopy height (height (max.))	collected from the literature, the BADM product, and or site principal investigators (Migliavacca et al. 2021)
		species richness	the number of upper story species of the forest, which was collected from the literature, the BADM product, and or site principal investigators

		rooting depth	extracted from a remote-sensing-based product (Stocker et al. 2023) at the corresponding 0.05°× 0.05° pixel for each site.
abundance-weighted plant hydraulic traits (Table S7)	The plant hydraulic traits and their diversity in forests have been found to play a critical role in land-atmosphere feedback during drought (Anderegg et al. 2018). Combining the species composition of each site and the species-specific traits from the TRY database (Kattge et al. 2020) allowed us to calculate the abundance-weighted mean and standard deviation of plant hydraulic traits. It needs to be noted that these abundance-weighted traits are only calculated in the sites where the proportion of species is greater than 50% based on the available information.	abundance-weighted mean wood density (wood density (mean))	
		abundance-weighted standard deviation of wood density (wood density (s.d.))	
		abundance-weighted mean P50 (P50 (mean))	the water potential at 50% loss of hydraulic conductivity
		abundance-weighted standard deviation of P50 (P50 (s.d.))	
		abundance-weighted mean HSM50 (HSM50 (mean))	the difference between P50 and the minimum water potential experienced
		abundance-weighted standard deviation of HSM50 (HSM50 (s.d.))	

Table S4. unconditional independence test results across all studied sites. KCIT (Kernel-based Conditional Independence test), HSIC (Hilbert-Schmidt independence criterion) gamma, and HSIC perm are independence test methods. Values indicate the p values of the tests whether legacy effects are **unconditionally** and significantly ($p<0.05$) dependent on one potential driver based on the corresponding methods. If at least two out of the three methods yield a p-value of less than 0.05, the potential driver was selected as a candidate driver. See Table S2 in the main text for the detailed definition of all potential drivers.

Potential drivers	KCIT	HSIC gamma	HSIC perm
Concurrent impact peak	0.027093	0.137549	0.138614
Concurrent effects	0.222805	0.248665	0.267327
Drought intensity	0.513845	0.631623	0.544554
Drought duration	0.232773	0.184186	0.178218
Drought timing	0.779238	0.858513	0.861386
Pre-drought water limitation	0.276609	0.788556	0.80198
Post-drought water limitation	0.425179	0.891263	0.871287
Aridity	0.524714	0.714602	0.732673
Mean temperature	0.346519	0.682394	0.633663
Precipitation seasonality	0.903551	0.898567	0.950495
Temperature seasonality	0.691294	0.756502	0.584158
**Forest vs non-forest**	**0.005683**	**0.007948**	**0.019802**

Table S5. Unconditional independence test results in forests. KCIT (Kernel-based Conditional Independence test), HSIC (Hilbert-Schmidt independence criterion) gamma, and HSIC perm are independence test methods. Values indicate the p values of the tests whether legacy effects are **unconditionally** and significantly ($p<0.05$) dependent on one potential driver based on the corresponding methods. If at least two out of the three methods yield a p-value of less than 0.05, the potential driver was selected as a candidate driver. See Table S2 in the main text for the detailed definition of all potential drivers.

Potential drivers	KCIT	HSIC gamma	HSIC perm
Concurrent impact peak	0.779863	0.678688	0.514851
Concurrent effects	0.452316	0.687723	0.564356
Drought intensity	0.420539	0.518261	0.405941
Drought duration	0.322582	0.714801	0.673267
Drought timing	0.712422	0.329207	0.237624
Pre-drought water limitation	0.0879	0.662263	0.613861
Post-drought water limitation	0.802103	0.924245	0.881188
aridity	0.339006	0.440504	0.29703
Mean temperature	0.836943	0.907891	0.920792
Precipitation seasonality	0.945251	0.775384	0.70297
Temperature seasonality	0.636617	0.77435	0.584158
Forest mean age	0.384362	0.704157	0.693069
Canopy maximum height	0.491415	0.948149	0.940594
Species richness	0.112001	0.487167	0.366337
Rooting depth	0.519021	0.177629	0.118812
Ecosystem-weighted mean wood density	0.401764	0.615977	0.50495
Ecosystem-weighted standard deviation of wood density	0.555718	0.526301	0.455446
**Ecosystem-weighted mean P50**	0.105626	**0.033247**	**0.029703**
Ecosystem-weighted standard deviation of P50	0.390636	0.767525	0.712871
Ecosystem-weighted mean HSM50	0.563171	0.539588	0.39604
Ecosystem-weighted standard deviation of HSM50	0.408629	0.642927	0.485149

Table S6. The forest mean age, canopy maximum height, and species richness at the forest sites detected significant drought legacy effects. The age, the time since management-induced disturbance, of all sites is normalized to the year 2017. Nsp is the number of upper-story species of each site. Plant functional types (PFT): DBF, deciduous broad-leaf forests; EBF, evergreen broad-leaf forests and ENF, Evergreen needle-leaf forests.

site	PFT	Mean age in 2017 (year)	Age source	Canopy maximum height (m)	Height source	Species richness (Nsp)	Species richness source
CA-LP1	ENF			15	(Migliavacca et al. 2021)	1	(Gomarasca et al. 2023)
CZ-RAJ	ENF	114	(McGloin et al. 2018)	33	(McGloin et al. 2018)	1	(Gomarasca et al. 2023)
DE-Hai	DBF	149	(Flechard et al. 2020)	35	(Migliavacca et al. 2021)	4	(Yu et al. 2022)
DE-Lnf	DBF				(Migliavacca et al. 2021)	1	(Yu et al. 2022)
FR-Hes	DBF	52	(Flechard et al. 2020)	16	(Migliavacca et al. 2021)	7	(Gomarasca et al. 2023)
FR-LBr	ENF	48	(Flechard et al. 2020)	21.9775	(Migliavacca et al. 2021)	21	(Gomarasca et al. 2023)
FR-Pue	EBF	76	(Flechard et al. 2020)	6.3684	(Migliavacca et al. 2021)	5	(Gomarasca et al. 2023)
IL-Yat	ENF	52	(Musavi et al. 2017)	10	(Migliavacca et al. 2021)	1	(Gomarasca et al. 2023)
IT-Lav	ENF	101	(Musavi et al. 2017)	36	(Marcolla, Pitacco, and Cescatti 2003)	3	(Gomarasca et al. 2023)
SE-Nor	ENF	119	(Flechard et al. 2020)	26	(Kovalets et al. 2018)(85)	7	(Gomarasca et al. 2023)
US-GLE	ENF	192	PI_BADM	17	(Migliavacca et al. 2021)	2	(Gomarasca et al. 2023)
US-Me2	ENF	106	PI_BADM	16.5	(Migliavacca et al. 2021)	3	(Gomarasca et al. 2023)
US-MMS	DBF	96	PI_BADM	27	(Migliavacca et al. 2021)	6	(Gomarasca et al. 2023)

Table S7. Abundance-weighted plant hydraulic traits at the forest sites detected significant drought legacy effects. Abundance-weighted mean and standard deviation of wood density, water potential at 50% loss of hydraulic conductivity (P50), and hydraulic safety margin (HSM50, as the difference between P50 and the minimum water potential experienced).

site	PFT	Abundance-weighted mean wood density	Abundance-weighted standard deviation of wood density	Abundance-weighted mean P50	Abundance-weighted standard deviation of P50	Abundance-weighted mean HSM50	Abundance-weighted standard deviation of HSM50
CA-LP1	ENF	0.407247	0	-3.34101	0	1.642115	0
CZ-RAJ	ENF	0.455955	0	-3.72645	4.46E-16	0.827778	0
DE-Hai	DBF	0.662495	0.042495	-2.64596	0.188205	0.689565	0.258971
DE-Lnf	DBF	0.682027	0	-2.64448	0	0.86	0
FR-Hes	DBF	0.676085	0.026748	-2.72802	0.350934	0.822897	0.184057
FR-LBr	ENF	0.495593	0.052487	-3.39621	0.093096	1.363782	0.071865
FR-Pue	EBF	0.782014	0.13771	-4.34707	2.22742	-0.60188	0.10267
IL-Yat	ENF	0.606944	0	-3.61045	4.46E-16	1.106667	0
IT-Lav	ENF	0.518292	0.070602	-3.58224	0.396025	-0.01983	0.568357
SE-Nor	ENF	0.485893	0.039944	-3.51596	0.30907	0.918708	0.12374
US-GLE	ENF	0.396346	0.062276	-4.31338	0.97337	1.83808	0
US-Ha1	DBF	0.516504	0.067431	-2.25572	0.402251	0.417027	0.165374
US-Me2	ENF	0.438479	5.58E-17	-2.8851	0.640773	0.466904	0
US-MMS	DBF	0.659797	0.26386	-2.59963	0.647001	-	-

References

Ammann, C. (2016). *FLUXNET2015 CH-Oe1 Oensingen grassland*. FluxNet; Agroscope Zuerich. https://doi.org/10.18140/FLX/1440135

Anderegg, W. R. L., Konings, A. G., Trugman, A. T., Yu, K., Bowling, D. R., Gabbitas, R., Karp, D. S., Pacala, S., Sperry, J. S., Sulman, B. N., & Zenes, N. (2018). Hydraulic diversity of forests regulates ecosystem resilience during drought. *Nature*, *561*(7724), 538–541. https://doi.org/10.1038/s41586-018-0539-7

Arain, M. A. (2016a). *FLUXNET2015 CA-TP1 Ontario—Turkey Point 2002 Plantation White Pine*. FluxNet; McMaster University. https://doi.org/10.18140/FLX/1440050

Arain, M. A. (2016b). *FLUXNET2015 CA-TP3 Ontario—Turkey Point 1974 Plantation White Pine*. FluxNet; McMaster University. https://doi.org/10.18140/FLX/1440052

Arndt, S. (2013). *Wombat State Forest OzFlux-tower site, OzFlux: Australian and New Zealand Flux Research and Monitoring*. OzFlux. https://doi.org/hdl:102.100.100/14237

Arriga, N., Goded, I., Manca, G., & ICOS Ecosystem Thematic Centre. (2022). *Warm winter 2020 ecosystem eddy covariance flux product from San Rossore 2*. ICOS Carbon Portal. https://doi.org/10.18160/EPYB-PSFM

Aurela, M., Tuovinen, J.-P., Hatakka, J., Lohila, A., Mäkelä, T., Rainne, J., & Lauria, T. (2016). *FLUXNET2015 FI-Sod Sodankyla*. FluxNet; Finnish Meteorological Institute. https://doi.org/10.18140/FLX/1440160

Baldocchi, D., & Ma, S. (2016). *FLUXNET2015 US-Ton Tonzi Ranch*. FluxNet; University of California, Berkeley. https://doi.org/10.18140/FLX/1440092

Berbigier, P., & Loustau, D. (2016). *FLUXNET2015 FR-LBr Le Bray*. FluxNet; INRA - UMR ISPA. https://doi.org/10.18140/FLX/1440163

Beringer, J. (2013a). *Daly Uncleared OzFlux tower site, OzFlux: Australian and New Zealand Flux Research and Monitoring*. OzFlux. https://doi.org/hdl:102.100.100/14239

Beringer, J. (2013b). *Howard Springs OzFlux tower site, OzFlux: Australian and New Zealand Flux Research and Monitoring*. OzFlux. https://doi.org/hdl:102.100.100/14234

Beringer, J. (2013c). *Sturt Plains OzFlux tower site, OzFlux: Australian and New Zealand Flux Research and Monitoring*. OzFlux. https://doi.org/hdl:102.100.100/14230

Beringer, J. (2013d). *Yanco JAXA OzFlux tower site, OzFlux: Australian and New Zealand Flux Research and Monitoring*. OzFlux. https://doi.org/hdl:102.100.100/14235

Bernhofer, C., Gruenwald, J. T., & ICOS Ecosystem Thematic Centre. (2022a). *Warm winter 2020 ecosystem eddy covariance flux product from Grillenburg*. ICOS Carbon Portal. https://doi.org/10.18160/A4NW-3VE1

Bernhofer, C., Gruenwald, J. T., & ICOS Ecosystem Thematic Centre. (2022b). *Warm winter 2020 ecosystem eddy covariance flux product from Tharandt*. ICOS Carbon Portal. https://doi.org/10.18160/8FBV-1K18

Besnard, S., Carvalhais, N., Arain, M. A., Black, A., Bruin, S. de, Buchmann, N., Cescatti, A., Chen, J., Clevers, J. G. P. W., Desai, A. R., Gough, C. M., Havrankova, K., Herold, M., Hörtnagl, L., Jung, M., Knohl, A., Kruijt, B., Krupkova, L., Law, B. E., … Reichstein, M. (2018). Quantifying the effect of forest age in annual net forest carbon balance. *Environmental Research Letters*, *13*(12), 124018. https://doi.org/10.1088/1748-9326/aaeaeb

Black, T. (2018). *AmeriFlux AmeriFlux CA-Obs Saskatchewan—Western Boreal, Mature Black Spruce*. AmeriFlux; The University of British Columbia. https://doi.org/10.17190/AMF/1375198

Black, T. (2021). *AmeriFlux FLUXNET-1F CA-LP1 British Columbia—Mountain pine beetle-attacked lodgepole pine stand*. AmeriFlux; University of British Columbia. https://doi.org/10.17190/AMF/1832155

Blanken, P., Monson, R., Burns, S., Bowling, D., & Turnipseed, A. (2022). *AmeriFlux FLUXNET-1F US-NR1 Niwot Ridge Forest (LTER NWT1)*. AmeriFlux; University of Colorado. https://doi.org/10.17190/AMF/1871141

Buchmann, N., Eugster, W., Hörtnagl, L., Fuchs, K., & ICOS Ecosystem Thematic Centre. (2022). *Warm winter 2020 ecosystem eddy covariance flux product from Fruebuel grassland*. ICOS Carbon Portal. https://doi.org/10.18160/4SAX-0ZYA

Buchmann, N., Eugster, W., Paul-Limoges, E., Gharun, M., & ICOS Ecosystem Thematic Centre. (2022). *Warm winter 2020 ecosystem eddy covariance flux product from Laegern*. ICOS Carbon Portal. https://doi.org/10.18160/51Z6-S5XF

Buchmann, N., Hörtnagl, L., Merbold, L., Gharun, M., & ICOS Ecosystem Thematic Centre. (2022). *Warm winter 2020 ecosystem eddy covariance flux product from Davos*. ICOS Carbon Portal. https://doi.org/10.18160/1JA9-VJEV

Buchmann, N., Merbold, L., Fuchs, K., Feigenwinter, I., & ICOS Ecosystem Thematic Centre. (2022). *Warm winter 2020 ecosystem eddy covariance flux product from Chamau grassland*. ICOS Carbon Portal. https://doi.org/10.18160/V5RX-VAWY

Cañete, E. P. S., Ortiz, P. S., Jiménez, M. R. M., Poveda, F. D., Priego, O. P., Ballesteros, A. L., & Kowalski, A. S. (2016). *FLUXNET2015 ES-LJu Llano de los Juanes*. FluxNet; University of Granada. https://doi.org/10.18140/FLX/1440157

Cleverly, J., Eamus, D., Faux, R., Grant, N. M., & Li, Z. (2021). *Alice Springs Mulga Flux Data Collection, Version 1.0 (Dataset)*. Terrestrial Ecosystem Research Network (TERN). https://doi.org/hdl:102.100.100/14217

Cremonese, E., Galvagno, M., Morra di Cella, U., Migliavacca, M., & ICOS Ecosystem Thematic Centre. (2022). *Warm winter 2020 ecosystem eddy covariance flux product from Torgnon*. ICOS Carbon Portal. https://doi.org/10.18160/9J7C-Q5QP

Cuntz, M., Longdoz, B., & ICOS Ecosystem Thematic Centre. (2022). *Warm winter 2020 ecosystem eddy covariance flux product from Hesse*. ICOS Carbon Portal. https://doi.org/10.18160/GFQX-22T7

Desai, A. (2016). *FLUXNET2015 US-PFa Park Falls/WLEF*. FluxNet; University of Wisconsin. https://doi.org/10.18140/FLX/1440089

Dufrêne, E., Berveiller, D., Delpierre, N., & ICOS Ecosystem Thematic Centre. (2022). *Warm winter 2020 ecosystem eddy covariance flux product from Fontainebleau-Barbeau*. ICOS Carbon Portal. https://doi.org/10.18160/X1J0-H684

Flechard, C. R., Ibrom, A., Skiba, U. M., de Vries, W., van Oijen, M., Cameron, D. R., Dise, N. B., Korhonen, J. F. J., Buchmann, N., Legout, A., Simpson, D., Sanz, M. J., Aubinet, M., Loustau, D., Montagnani, L., Neirynck, J., Janssens, I. A., Pihlatie, M., Kiese, R., … Sutton, M. A. (2020). Carbon–nitrogen interactions in European forests and semi-natural vegetation – Part 1: Fluxes and budgets of carbon, nitrogen and greenhouse gases from ecosystem monitoring and modelling. *Biogeosciences*, *17*(6), 1583–1620. https://doi.org/10.5194/bg-17-1583-2020

Gianelle, D., Belelli Marchesini, L., Sottocornola, M., & ICOS Ecosystem Thematic Centre. (2022). *Warm winter 2020 ecosystem eddy covariance flux product from Lavarone*. ICOS Carbon Portal. https://doi.org/10.18160/HZSQ-G19C

Goldstein, A. (2016). *FLUXNET2015 US-Blo Blodgett Forest*. FluxNet; University of California, Berkeley. https://doi.org/10.18140/FLX/1440068

Gomarasca, U., Migliavacca, M., Kattge, J., Nelson, J. A., Niinemets, Ü., Wirth, C., Cescatti, A., Bahn, M., Nair, R., Acosta, A. T. R., Arain, M. A., Beloiu, M., Black, T. A., Bruun, H. H., Bucher, S. F., Buchmann, N., Byun, C., Carrara, A., Conte, A., … Reichstein, M. (2023). Leaf-level coordination principles propagate to the ecosystem scale. *Nature Communications*, *14*(1), 1. https://doi.org/10.1038/s41467-023-39572-5

Gruening, C., Goded, I., Cescatti, A., Manca, G., & Seufert, G. (2016). *FLUXNET2015 IT-SRo San Rossore*. FluxNet; European Commission - Joint Research Centre. https://doi.org/10.18140/FLX/1440176

Hollinger, D. (2022). *AmeriFlux FLUXNET-1F US-Ho2 Howland Forest (west tower)*. AmeriFlux; USDA Forest Service. https://doi.org/10.17190/AMF/1881581

Ibrom, A., & Pilegaard, K. (2016). *FLUXNET2015 DK-Sor Soroe*. FluxNet; Technical University of Denmark (DTU). https://doi.org/10.18140/FLX/1440155

Janssens, I., De Meulder, T., Roland, M., Segers, J., & ICOS Ecosystem Thematic Centre. (2022). *Warm winter 2020 ecosystem eddy covariance flux product from Brasschaat*. ICOS Carbon Portal. https://doi.org/10.18160/YVBQ-K6WF

Kattge, J., Bönisch, G., Díaz, S., Lavorel, S., Prentice, I. C., Leadley, P., Tautenhahn, S., Werner, G. D. A., Aakala, T., Abedi, M., Acosta, A. T. R., Adamidis, G. C., Adamson, K., Aiba, M., Albert, C. H., Alcántara, J. M., Alcázar C, C., Aleixo, I., Ali, H., … Wirth, C. (2020). TRY plant trait database – enhanced coverage and open access. *Global Change Biology*, *26*(1), 119–188. https://doi.org/10.1111/gcb.14904

Knohl, A., Siebicke, L., Tiedemann, F., Kolle, O., & ICOS Ecosystem Thematic Centre. (2022). *Warm winter 2020 ecosystem eddy covariance flux product from Hainich*. ICOS Carbon Portal. https://doi.org/10.18160/CR66-PJ24

Knohl, A., Tiedemann, F., Kolle, O., Schulze, E.-D., Anthoni, P., Kutsch, W., Herbst, M., & Siebicke, L. (2016). *FLUXNET2015 DE-Lnf Leinefelde*. FluxNet; University of Goettingen, Bioclimatology. https://doi.org/10.18140/FLX/1440150

Kovalets, I., Avila, R., Mölder, M., Kovalets, S., & Lindroth, A. (2018). Verification of a One-Dimensional Model of $$\hbox {CO}_{2}$$Atmospheric Transport Inside and Above a Forest Canopy Using Observations at the Norunda Research Station. *Boundary-Layer Meteorology*, *168*(1), 103–126. https://doi.org/10.1007/s10546-018-0340-z

Law, B. (2022). *AmeriFlux FLUXNET-1F US-Me2 Metolius mature ponderosa pine*. AmeriFlux; Oregon State University. https://doi.org/10.17190/AMF/1854368

Liddell, M. (2013a). *Cape Tribulation OzFlux tower site, OzFlux: Australian and New Zealand Flux Research and Monitoring*. OzFlux. https://doi.org/hdl:102.100.100/14242

Liddell, M. (2013b). *Robson Creek OzFlux tower site, OzFlux: Australian and New Zealand Flux Research and Monitoring*. OzFlux. https://doi.org/hdl:102.100.100/14243

Litvak, M. (2021). *AmeriFlux FLUXNET-1F US-Mpj Mountainair Pinyon-Juniper Woodland*. AmeriFlux; University of New Mexico. https://doi.org/10.17190/AMF/1832161

Litvak, M. (2022). *AmeriFlux FLUXNET-1F US-Wjs Willard Juniper Savannah, Ver. 3-5, AmeriFlux AMP, (Dataset)*. AmeriFlux Management Project (AMP). https://doi.org/10.17190/AMF/1871146

Litvak, M. (2023a). *AmeriFlux FLUXNET-1F US-Seg Sevilleta grassland*. AmeriFlux; University of New Mexico. https://doi.org/10.17190/AMF/1984572

Litvak, M. (2023b). *AmeriFlux FLUXNET-1F US-Ses Sevilleta shrubland*. AmeriFlux; University of New Mexico. https://doi.org/10.17190/AMF/1984573

Ma, S., Xu, L., Verfaillie, J., & Baldocchi, D. (2023). *AmeriFlux FLUXNET-1F US-Var Vaira Ranch- Ione, Ver. 3-5, AmeriFlux AMP, (Dataset)*. AmeriFlux Management Project (AMP). https://doi.org/10.17190/AMF/1993904

Macfarlane, C. (2013). *Great Western Woodlands OzFlux: Australian and New Zealand Flux Research and Monitoring*. OzFlux. https://doi.org/hdl:102.100.100/14226

Mammarella, I., Vesala, T., Kolari, P., & ICOS Ecosystem Thematic Centre. (2022). *Warm winter 2020 ecosystem eddy covariance flux product from Hyytiälä*. ICOS Carbon Portal. https://doi.org/10.18160/XTBV-XCJV

Marcolla, B., Pitacco, A., & Cescatti, A. (2003). Canopy Architecture and Turbulence Structure in a Coniferous Forest. *Boundary-Layer Meteorology*, *108*(1), 39–59. https://doi.org/10.1023/A:1023027709805

Margolis, H. (2023). *AmeriFlux FLUXNET-1F CA-Qfo Quebec—Eastern Boreal, Mature Black Spruce*. AmeriFlux; Université Laval. https://doi.org/10.17190/AMF/2006960

Massman, B. (2022). *AmeriFlux FLUXNET-1F US-GLE GLEES*. AmeriFlux; USDA Forest Service. https://doi.org/10.17190/AMF/1871136

Matamala, R. (2016). *FLUXNET2015 US-IB2 Fermi National Accelerator Laboratory- Batavia (Prairie site)*. FluxNet; Argonne National Laboratory. https://doi.org/10.18140/FLX/1440072

McCaughey, H. (2022). *AmeriFlux FLUXNET-1F CA-Gro Ontario—Groundhog River, Boreal Mixedwood Forest*. AmeriFlux; Queen’s University. https://doi.org/10.17190/AMF/1902823

McGloin, R., Šigut, L., Havránková, K., Dušek, J., Pavelka, M., & Sedlák, P. (2018). Energy balance closure at a variety of ecosystems in Central Europe with contrasting topographies. *Agricultural and Forest Meteorology*, *248*, 418–431. https://doi.org/10.1016/j.agrformet.2017.10.003

Migliavacca, M., Musavi, T., Mahecha, M. D., Nelson, J. A., Knauer, J., Baldocchi, D. D., Perez-Priego, O., Christiansen, R., Peters, J., Anderson, K., Bahn, M., Black, T. A., Blanken, P. D., Bonal, D., Buchmann, N., Caldararu, S., Carrara, A., Carvalhais, N., Cescatti, A., … Reichstein, M. (2021). The three major axes of terrestrial ecosystem function. *Nature*, 1–5. https://doi.org/10.1038/s41586-021-03939-9

Mölder, M., Lankreijer, H., Lagergren, F., Holst, J., & ICOS Ecosystem Thematic Centre. (2022). *Warm winter 2020 ecosystem eddy covariance flux product from Norunda*. ICOS Carbon Portal. https://doi.org/10.18160/87R1-0HRM

Montagnani, L., Stecher, M., & ICOS Ecosystem Thematic Centre. (2022). *Warm winter 2020 ecosystem eddy covariance flux product from Renon*. ICOS Carbon Portal. https://doi.org/10.18160/WMCA-8P4P

Moors, E., & Elbers, J. (2016). *FLUXNET2015 NL-Loo Loobos*. FluxNet; ALTERRA / Wageningen Environmental Research. https://doi.org/10.18140/FLX/1440178

Munger, J. (2022). *AmeriFlux FLUXNET-1F US-Ha1 Harvard Forest EMS Tower (HFR1)*. AmeriFlux; Harvard University. https://doi.org/10.17190/AMF/1871137

Musavi, T., Migliavacca, M., Reichstein, M., Kattge, J., Wirth, C., Black, T. A., Janssens, I., Knohl, A., Loustau, D., Roupsard, O., Varlagin, A., Rambal, S., Cescatti, A., Gianelle, D., Kondo, H., Tamrakar, R., & Mahecha, M. D. (2017). Stand age and species richness dampen interannual variation of ecosystem-level photosynthetic capacity. *Nature Ecology & Evolution*, *1*(2), 2. https://doi.org/10.1038/s41559-016-0048

Novick, K., & Phillips, R. (2022). *AmeriFlux FLUXNET-1F US-MMS Morgan Monroe State Forest*. AmeriFlux; Indiana University. https://doi.org/10.17190/AMF/1854369

Ourcival, J.-M. (2016). *FLUXNET2015 FR-Pue Puechabon*. FluxNet; CNRS. https://doi.org/10.18140/FLX/1440164

Papale, D., Tirone, G., Valentini, R., Arriga, N., Belelli, L., Consalvo, C., Dore, S., Manca, G., Mazzenga, F., Sabbatini, S., & Stefani, P. (2016). *FLUXNET2015 IT-Ro2 Roccarespampani 2*. FluxNet; University of Tuscia - Vietrbo. https://doi.org/10.18140/FLX/1440175

Poveda, F. D., Perez-Priego, O., Sanchez-Canete, E. P., Lopez Ballesteros, A., & ICOS Ecosystem Thematic Centre. (2022). *Warm winter 2020 ecosystem eddy covariance flux product from Aguamarga*. ICOS Carbon Portal. https://doi.org/10.18160/3V53-5P08

Richardson, A., & Hollinger, D. (2023). *AmeriFlux FLUXNET-1F US-Bar Bartlett Experimental Forest*. AmeriFlux; Northern Arizona University. https://doi.org/10.17190/AMF/2006969

Schmidt, M., Graf, A., Dolfus, D., & ICOS Ecosystem Thematic Centre. (2022). *Warm winter 2020 ecosystem eddy covariance flux product from Rollesbroich*. ICOS Carbon Portal. https://doi.org/10.18160/Q02V-HAN4

Schmidt, M., Graf, A., Drüe, C., Dolfus, D., & ICOS Ecosystem Thematic Centre. (2022). *Warm winter 2020 ecosystem eddy covariance flux product from Wustebach*. ICOS Carbon Portal. https://doi.org/10.18160/JX9W-FBEJ

Scott, R. (2023a). *AmeriFlux FLUXNET-1F US-Whs Walnut Gulch Lucky Hills Shrub, Ver. 3-5, AmeriFlux AMP, (Dataset)*. AmeriFlux Management Project (AMP). https://doi.org/10.17190/AMF/1984574

Scott, R. (2023b). *AmeriFlux FLUXNET-1F US-Wkg Walnut Gulch Kendall Grasslands, Ver. 3-5, AmeriFlux AMP, (Dataset)*. AmeriFlux Management Project (AMP). https://doi.org/10.17190/AMF/1984575

Sigut, L., Foltýnová, L., Czerný, R., Pavelka, M., & ICOS Ecosystem Thematic Centre. (2022a). *Warm winter 2020 ecosystem eddy covariance flux product from Rajec*. ICOS Carbon Portal. https://doi.org/10.18160/AAYD-C9FK

Sigut, L., Foltýnová, L., Czerný, R., Pavelka, M., & ICOS Ecosystem Thematic Centre. (2022b). *Warm winter 2020 ecosystem eddy covariance flux product from Stitna*. ICOS Carbon Portal. https://doi.org/10.18160/0JY0-ZCD6

Sigut, L., Foltýnová, L., Kowalska, N., Pavelka, M., & ICOS Ecosystem Thematic Centre. (2022). *Warm winter 2020 ecosystem eddy covariance flux product from Bily Kriz forest*. ICOS Carbon Portal. https://doi.org/10.18160/2X51-1SD0

Silberstein, R. (2015). *Gingin OzFlux: Australian and New Zealand Flux Research and Monitoring*. OzFlux. https://doi.org/hdl:102.100.100/22677

Simioni, G., Marloie, O., Guibal, F., & ICOS Ecosystem Thematic Centre. (2022). *Warm winter 2020 ecosystem eddy covariance flux product from Font-Blanche*. ICOS Carbon Portal. https://doi.org/10.18160/KGN6-K1CX

Spano, D., Duce, P., Marras, S., Sirca, C., Arca, A., Zara, P., & Ventura, A. (2016). *FLUXNET2015 IT-Noe Arca di Noe—Le Prigionette*. FluxNet; University of Sassari; CNR-Ibimet Sassari. https://doi.org/10.18140/FLX/1440171

Stocker, B. D., Tumber-Dávila, S. J., Konings, A. G., Anderson, M. C., Hain, C., & Jackson, R. B. (2023). Global patterns of water storage in the rooting zones of vegetation. *Nature Geoscience*, *16*(3), 3. https://doi.org/10.1038/s41561-023-01125-2

Tech, C. (2013). *Calperum Chowilla OzFlux tower site, OzFlux: Australian and New Zealand Flux Research and Monitoring*. OzFlux. https://doi.org/hdl:102.100.100/14236

Valentini, R., Dore, S., Mazzenga, F., Sabbatini, S., Stefani, P., Tirone, G., & Papale, D. (2016). *FLUXNET2015 IT-Cpz Castelporziano*. FluxNet; University of Tuscia - Vietrbo. https://doi.org/10.18140/FLX/1440168

Varlagin, A., Kurbatova, J., Vygodskaya, N., & ICOS Ecosystem Thematic Centre. (2022). *Warm winter 2020 ecosystem eddy covariance flux product from Fyodorovskoye*. ICOS Carbon Portal. https://doi.org/10.18160/XMER-D4NR

Vincke, C., Heinesch, B., Longdoz, B., & ICOS Ecosystem Thematic Centre. (2022). *Warm winter 2020 ecosystem eddy covariance flux product from Vielsalm*. ICOS Carbon Portal. https://doi.org/10.18160/DF9X-QMRK

Wohlfahrt, G., Hammerle, A., & Hörtnagl, L. (2016). *FLUXNET2015 AT-Neu Neustift*. FluxNet; University of Innsbruck. https://doi.org/10.18140/FLX/1440121

Wood, J., & Gu, L. (2022). *AmeriFlux FLUXNET-1F US-MOz Missouri Ozark Site*. AmeriFlux; Oak Ridge National Laboratory; University of Missouri. https://doi.org/10.17190/AMF/1854370

Woodgate, W. (2013). *Tumbarumba OzFlux tower site, OzFlux: Australian and New Zealand Flux Research and Monitoring*. OzFlux. https://doi.org/hdl:102.100.100/14241

Yakir, D., Muller, J., Schwartz, E., Rotenberg, E., & ICOS Ecosystem Thematic Centre. (2022). *Warm winter 2020 ecosystem eddy covariance flux product from Yatir*. ICOS Carbon Portal. https://doi.org/10.18160/MAGT-CWRW

Yu, X., Orth, R., Reichstein, M., Bahn, M., Klosterhalfen, A., Knohl, A., Koebsch, F., Migliavacca, M., Mund, M., Nelson, J. A., Stocker, B. D., Walther, S., & Bastos, A. (2022). Contrasting drought legacy effects on gross primary productivity in a mixed versus pure beech forest. *Biogeosciences*, *19*(17), 4315–4329. https://doi.org/10.5194/bg-19-4315-2022
